# A new family of rolling-circle replication endonucleases widespread in archaeal viruses and plasmids

**DOI:** 10.1093/nar/gkag363

**Published:** 2026-04-24

**Authors:** Yixuan Wang, Zihao Duan, Zhao Chen, Shuyu Li, Huiyi Chen, Jiangling Chen, Yangyang Wang, Jiaying Wang, Kang An, Jialin Xiang, Shishen Du, Ying Liu, Mart Krupovic, Xiangdong Chen

**Affiliations:** State Key laboratory of Virology, College of Life Sciences, Wuhan University, 430072, China; State Key laboratory of Virology, College of Life Sciences, Wuhan University, 430072, China; State Key laboratory of Virology, College of Life Sciences, Wuhan University, 430072, China; State Key laboratory of Virology, College of Life Sciences, Wuhan University, 430072, China; State Key laboratory of Virology, College of Life Sciences, Wuhan University, 430072, China; State Key laboratory of Virology, College of Life Sciences, Wuhan University, 430072, China; State Key laboratory of Virology, College of Life Sciences, Wuhan University, 430072, China; State Key laboratory of Virology, College of Life Sciences, Wuhan University, 430072, China; State Key laboratory of Virology, College of Life Sciences, Wuhan University, 430072, China; State Key laboratory of Virology, College of Life Sciences, Wuhan University, 430072, China; State Key laboratory of Virology, College of Life Sciences, Wuhan University, 430072, China; State Key laboratory of Virology, College of Life Sciences, Wuhan University, 430072, China; Institut Pasteur, Université Paris Cité, CNRS UMR6047, Cell Biology and Virology of Archaea Unit, 75015 Paris, France; State Key laboratory of Virology, College of Life Sciences, Wuhan University, 430072, China

## Abstract

Archaea possess a remarkably diverse mobilome, but for many archaeal viruses and plasmids, even the basic processes, such as genome replication, remain poorly understood. Here, we characterize a previously uncharacterized family of putative rolling-circle replication endonucleases, termed Rep-Arvir, widespread among viruses and plasmids associated with phylogenetically diverse archaea, including halophiles, methanogens, and hyperthermophiles. We show that RepSNJ2, encoded by the temperate pleomorphic virus SNJ2, a model member of the *Pleolipoviridae* family, is essential for driving autonomous replication. Moreover, a conserved hairpin-forming DNA element downstream of *repsnj2* likely functions as the recognition site for RepSNJ2 and origin of replication of SNJ2. Notably, the functional replication operon is restored only following the excision and circularization of the SNJ2 viral genome, representing an elegant regulatory mechanism controlling the lysogeny-replication switch. Leveraging this system, we constructed SNJ2-based shuttle vectors that enable stable gene expression and are compatible with other *Natrinema* plasmids. Structural modeling revealed that the Rep-Arvir family is distantly related to the bacterial Rep_trans family endonucleases, a relationship not recognizable at the sequence level. These findings provide evidence for a previously unrecognized replication mechanism in archaea, highlight deep evolutionary links between archaeal and bacterial replicons, and provide a versatile genetic platform for studying virus–host interactions in hypersaline environments.

## Graphical Abstract

**Graphical Abstract:**
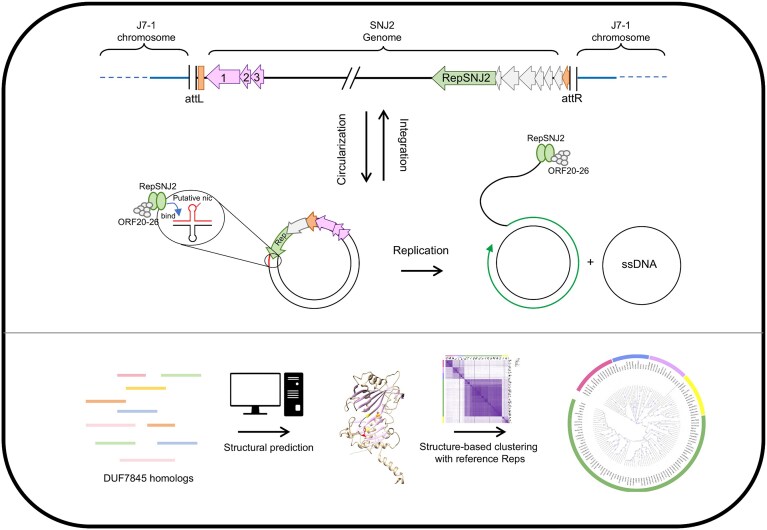

## Introduction

Viruses are ubiquitous and exceptionally diverse, playing essential roles in shaping the dynamics of microbial ecosystems and driving horizontal gene transfer in all domains of life [1]. Over evolutionary timescales, viruses have evolved a wide spectrum of genome replication strategies. This diversity is dictated by the specific genome type (e.g. DNA versus RNA, single- versus double-stranded, linear versus circular) and the extent to which they rely on the host’s replication machinery [2–6]. Deciphering these replication mechanisms is crucial for understanding viral life cycles and for developing both antiviral and synthetic biology applications.

Rolling-circle replication (RCR) is one of the most common replication mechanisms used by plasmids and small viral genomes. Two nonhomologous, structurally distinct groups of RCR initiation endonucleases have been discovered [7]. Endonucleases of the HUH superfamily adopt the structural fold known as the RNA-recognition motif, also found in the palm domain polymerases [8], whereas Rep_trans family endonucleases adopt a distinct fold also found in the TATA-binding protein (TBP) [9]. Nevertheless, both types of endonucleases initiate replication by similar mechanisms, involving a conserved catalytic tyrosine residue, which nicks the double-stranded DNA within the origin of replication (*ori*) site, forming a covalent phosphodiester bond with the 5′-phosphate and releasing the 3′-OH, which serves as a primer for unidirectional DNA synthesis [9]. The HUH superfamily members have been discovered in mobile genetic elements (MGEs) from all three domains of life [10, 11]. By contrast, Rep_trans enzymes appear to be restricted to bacteria, suggesting that this family of endonucleases has emerged within the bacterial domain following its divergence from the common ancestor with archaea. The RCR mechanism has been extensively studied in bacteria and certain eukaryotic viruses with single-stranded DNA (ssDNA) genomes [12, 13]. By contrast, the RCR landscape in archaeal viruses remains largely unexplored, with a notable exception of a few experimental studies in hyperthermophilic and halophilic archaea where HUH superfamily enzymes were shown to be involved in the replication of plasmids and viruses [14–18].

Archaeal viruses represent one of the most enigmatic branches of the virosphere, notable for their morphological diversity and the frequent absence of recognizable replication proteins [19]. Although recent metagenomic surveys have greatly expanded the diversity of known archaeal viral genomes [20], only a small subset has had their replication strategies experimentally characterized [17, 21–26]. Many of these viruses lack identifiable DNA polymerases or replication initiators, implying either a complete reliance on host machinery or the existence of entirely novel replication elements yet to be discovered [27, 28].

The *Pleolipoviridae* family comprises pleomorphic viruses infecting extremely halophilic archaea [29]. These viruses possess relatively small DNA genomes (~7–16 kb) with variable topology (circular single- or double-stranded, or linear double-stranded) and nonlytic life cycles. Pleolipoviruses represent one of the most prevalent haloarchaeal virus groups, detected across all major genera of halophilic archaea, and are commonly found as proviruses integrated in the genomes of their hosts [30]. These viruses play an important role in hypersaline ecosystems by engaging in complex symbiotic relationships with other co-infecting viruses and the host cells [31], modulating the cellular biofilm formation, motility and stress resistance, and CRISPR RNA expression [31, 32]. Viruses evolutionarily related to pleolipoviruses have been also described in hyperthermophilic and methanogenic archaea [33, 34], and in nano-sized symbiotic archaea of the phylum *Nanobdellati* [35], suggesting a long-standing coevolution of this virus lineage with archaeal hosts.

Family *Pleolipoviridae* comprises three officially recognized genera, *Alphapleolipovirus, Betapleolipovirus*, and *Gammapleolipovirus*, which roughly reflect the predicted replication strategies used by the member viruses [36]. Members of *Alphapleolipovirus* are predicted to replicate via a rolling-circle mechanism using virus-encoded initiators of the HUH superfamily [27, 37], whereas gammapleolipoviruses employ a protein-primed DNA polymerase for the replication of their linear genomes [38]. By contrast, no recognizable replication protein gene was identified in members of the *Betapleolipovirus* genus. However, in addition to the conserved core gene block shared by all pleolipoviruses, betapleolipoviruses uniquely possess two additional conserved open reading frames (ORFs) [29]. One of these ORFs, exemplified by ORF19 of SNJ2, encodes a putative protein with a winged helix-turn-helix (wHTH) DNA-binding domain, which has been proposed to functionally replace the RCRE found in alphapleolipoviruses [27]. However, this ORF shows no detectable homology to known replication proteins, making its function impossible to infer by conventional sequence-based approaches [27, 29, 39].

SNJ2, a temperate virus isolated from *Natrinema* sp. J7-1, represents a genetically tractable model for *Betapleolipovirus* replication. It is the only known pleomorphic archaeal virus with a fully characterized integration-excision module that regulates its lysogeny-replication transition [40, 41]. However, despite extensive genetic and molecular studies, SNJ2 replication mechanism has remained unknown. Here, we address this long-standing question by integrating genetic, structural modeling, and functional analyses to provide a mechanistic model for the replication strategy of SNJ2 and, by extension, that of other betapleolipoviruses. We identify ORF19 as a previously uncharacterized RCR initiator, Rep-SNJ2, which defines a new, structurally conserved family of RCR endonucleases, termed Rep-Arvir, distinct from the HUH superfamily, but distantly related to the bacterial Rep_trans family. Furthermore, we uncover an essential downstream DNA hairpin structure that serves as the viral origin of replication, providing a *cis-*acting DNA element specifically recognized by Rep-SNJ2. Finally, we show that Rep-Arvir family extends far beyond haloarchaeal pleolipoviruses to include morphologically and evolutionarily distinct groups of viruses and plasmids from diverse archaea. Together, these findings provide the first mechanistic framework for genome replication initiation by Rep-Arvir family enzymes and provide a versatile shuttle vector for archaeal genetics, offering new insight into the modular evolution of viruses and other MGEs.

## Materials and methods

### Strains, media, growth conditions, and transformation

All strains and plasmids used in this study are listed in Supplementary Tables S7 and S8 in the supplemental material. The detailed procedures for strain and plasmid construction are provided in the Supplementary “Materials and methods.” *Natrinema* sp. J7 and other haloarchaeal strains were cultured in Halo-2 medium or 23% modified growth medium (MGM) at 45°C, as previously described [42]. Agar plates were prepared with 15 g/l Bacto Agar (BD). The Halo-2 medium consisted of 250 g NaCl, 30 g MgCl_2_·6H_2_O, 2.5 g lactalbumin hydrolysate (Difco Laboratories), and 2 g Bacto yeast extract (Difco Laboratories) per liter of water. Casamino Acids medium (Hv-Ca, Minimal Medium) and MGM were prepared according to previously published protocols [42]. The Hv-Ca medium contained 144 g NaCl, 18 g MgCl_2_·6H_2_O, 21 g MgSO_4_·7H_2_O, 4.2 g KCl, 5 g Amicase (Sigma), 0.5 g CaCl_2_, and 30 ml of 1 M Tris–HCl (pH 7.5) per liter of water. This liquid minimal medium was used to culture the transformants. For the selection and isolation of these transformants, cells were plated onto solid agar medium (referred to as MM plates), which was prepared by supplementing the liquid MM with 1.5% (w/v) agar. The 23% MGM contained 184 g NaCl, 23 g MgCl_2_·6H_2_O, 26.8 g MgSO_4_·7H_2_O, 5.4 g KCl, 5 g peptone (Difco Laboratories), 3 g Bacto yeast extract (Difco Laboratories), 0.5 g CaCl_2_, and 30 ml of 1 M Tris–HCl (pH 7.5) per liter of water. When required, 5-fluoroorotic acid (5-FOA) was added to a final concentration of 0.04 mg/ml, and mevinolin (Mev) was added at 5 μg/ml to cultures in 23% MGM. *Escherichia coli* strains were grown at 37°C in Luria–Bertani medium, supplemented with ampicillin (100 μg/ml) or kanamycin (50 μg/ml) as needed.

Transformation of haloarchaea, including *Natrinema* sp. J7 and other strains, was performed using a modified polyethylene glycol method, as described previously [43, 44]. *Escherichia coli* strains DH5α, JM110, and BL21 were transformed using the CaCl₂ method [45]. Standard molecular manipulations in *E. coli* were carried out as described previously [46].

### Analysis of co-transcription and transcription start sites

For analysis of *orf19*-*26* co-transcription, total RNA from J7-1 was extracted using the TRIzol reagent (Invitrogen) as previously described [31]. For reverse transcription-PCR, residual genomic DNA was removed using the gDNA Eraser (TaKaRa), and complementary DNA (cDNA) was synthesized from 5 µg of total RNA using random primers (RT reagent kit; TaKaRa). The synthesized cDNA served as the template for transcription analysis, employing specific primers (Supplementary Table S9) designed to amplify the intragenic and intergenic regions of *orf19*-*orf26*.

To determine the 5′- and 3′-ends of the transcript, RNA circularization and extraction were performed following the protocol outlined in a previous study [47]. In brief, purified self-ligated RNA was treated with gDNA Eraser to remove any remaining genomic DNA, followed by reverse transcription. The cDNA of the 5′-3′ ligated RNA was then amplified using gene-specific primer pairs, with a subsequent polymerase chain reaction (PCR) using nested primer (supplementary data is available at Supplementary Table S9). The use of nested PCR significantly improved amplification specificity and reduced the likelihood of false-positive fragments from the first PCR reaction. Finally, the specific nested PCR products spanning the 5′–3′ junction were purified, ligated into a T-vector, and transformed into *E. coli*. Plasmids extracted from positive clones were then analyzed by Sanger sequencing to precisely pinpoint the transcription start site.

### Western blot analysis

To analyze protein expression, plasmids expressing either an ORF26-GFP fusion or ORF19-His variants (wild type and active-site mutants) under the control of the P promoter were generated (detailed plasmid construction procedures and primers are provided in the supplementary materials and Supplementary Table S9). These plasmids were transformed into the J7-1-F and J7-3-F strains, respectively. The transformants harboring pFJ6-P-ORF26-GFP or pFJ6-P-ORF19-His variants were cultured to the mid-exponential phase (OD_600_ = 0.6–0.8) in Halo-2 medium. Cells were harvested by centrifugation. For sample preparation, different denaturation strategies were employed based on the target proteins. For the ORF26-GFP samples, cell pellets were directly resuspended in sodium dodecyl sulfate–polyacrylamide gel electrophoresis (SDS–PAGE) sample buffer (HY-C-XP255; HY CEZMBIO, Wuhan, China) and boiled at 95°C for 10 min. In contrast, for the ORF19-His variants, cell pellets were resuspended in a buffer containing 8 M urea and disrupted by sonication (3–5 cycles) to ensure complete cell lysis and DNA shearing. This chemical denaturation approach strictly avoided high-temperature boiling to prevent potential heat-induced protein aggregation. Subsequently, the protein samples were resolved by 12% SDS–PAGE. Proteins were then transferred onto a nitrocellulose membrane (Vazyme, Nanjing, China) using a wet electroblotting system (Bio-Rad, Hercules, CA, USA) in transfer buffer (HYWBX120; HY CEZMBIO, Wuhan, China). For the ORF19-His samples, the membranes were reversibly stained with 1× Ponceau S solution (P0012, Solarbio, Beijing, China) prior to blocking to serve as a total protein loading control. Membranes were then washed 3× 5 min with TBST and used for immunoblotting.

For immunoblotting, the membranes were blocked with 5% (w/v) skim milk in TBST for 1 h at room temperature. The detection strategies were then tailored to the specific fusion tags. For ORF26-GFP detection, the membrane was incubated with a mouse anti-GFP monoclonal antibody (AE012, ABclonal, Wuhan, China; dilution 1:10 000) at 4°C overnight. Following three 10-min washes with TBST, the membrane was subsequently incubated with an HRP-conjugated goat anti-mouse secondary antibody (AS003, ABclonal, Wuhan, China; dilution 1:10 000) for 1 h at room temperature. Conversely, for ORF19-His detection, the membrane was directly incubated with an HRP-conjugated anti-His antibody (GNI4310HS, GNI Mission, Shanghai, China; dilution 1:10 000) for 1 h at room temperature. After three final washes with TBST, the protein signals for all blots were visualized using the ECL Super Kit (RM02867, ABclonal, Wuhan, China) and recorded with a ChemiDoc MP Imaging System (Bio-Rad).

### Quantification of viral genome, attP, and vectors

The presence of the circular SNJ2 genome was detected by standard PCR using primers *attP*-F and *attP*-R. The amplification was performed in a 20-μl reaction volume using the P222 DNA Polymerase (Vazyme, Nanjing, China) according to the manufacturer’s instructions.

The relative copy number of the SNJ2 genome or *attP* was quantified using quantitative PCR (qPCR), with cell cultures treated with or without mitomycin C (MMC) induction as templates. Briefly, cultures were grown to mid-exponential phase (OD_600_ = 0.6–0.8) in Halo-2 medium and then treated with MMC (1 µg/ml) for 30 min. Cells were harvested by centrifugation, washed twice with an equal volume of Halo-2 to remove MMC, and resuspended in Halo-2 medium. Samples were taken at various time points post-induction. To minimize the inhibitory effects of high salt concentrations on qPCR, 10 µl of cell suspension was diluted in 490 µl of distilled water, leading to rapid cell lysis. The single-copy *radA* gene located on the host chromosome served as the reference, while *orf7* and *attP* of SNJ2 were used as markers for the viral genome. To assess the plasmid-to-chromosome copy number ratio, we used vector-specific primers (vector-F/R). The primers used for qPCR are listed in Supplementary Table S9. The qPCR reaction was performed in a 20 µl mixture containing 5 µl of template DNA, 10 µl of iTaq Universal SYBR Green Supermix (Bio-Rad), 1 µl of primer pairs (10 µM), and 4 µl of distilled water. The amplification was carried out on a CFX96 Connect Real-Time PCR Detection System (Bio-Rad). The thermocycling conditions consisted of an initial denaturation at 95°C for 5 min, followed by 40 cycles of denaturation at 95°C for 5 s, and combined primer annealing and extension at 60°C for 30 s. A melt curve analysis was performed post-amplification to confirm primer specificity. Amplification efficiencies for all primer pairs were determined using standard curves and are detailed in Supplementary Table S9. The relative quantitative data were analyzed using the 2^−ΔCT^ method. Three independent experiments were conducted, with error bars representing standard deviations.

### Plasmid rescue and restriction digestion analysis

To verify the autonomous and episomal replication of the engineered plasmids in archaea, a plasmid rescue assay was performed. Total DNA was extracted from the archaeal transformants harboring pSNJ2Int^Mut^ or its derivatives using a genomic DNA extraction kit (Tiangen, Beijing, China). The extracted total DNA was then transformed into *E. coli* DH5α competent cells. Rescued plasmids were isolated from the resulting *E. coli* transformants using a plasmid miniprep kit (Tiangen, Beijing, China) and subsequently subjected to restriction enzyme digestion with *Stu*I and *Hind*III (Thermo Fisher Scientific, Waltham, MA, USA). The restriction digestion profiles were resolved by electrophoresis on a 1% agarose gel to confirm the structural integrity of the rescued plasmids. Furthermore, to definitively confirm their biological functionality and nonintegrated state, the plasmids re-isolated from *E. coli* were successfully transformed back into haloarchaeal cells, definitively ruling out chromosomal integration or structural rearrangements.

### Amylase activity assay

Amylase activity on plates was assessed following the method of Coronado *et al*. [48]. Transformants carrying pSNJ2^Mut^P3-19-Hpro-amyH were cultured in 5 ml of 20% HV-Ca medium until reaching the logarithmic phase. A 2-µl aliquot of the culture was then spotted onto 20% HV-Ca plates containing 2% (wt/vol) soluble starch. After incubating at 45°C for ~2 days, the plates were treated with a 0.3% I_2_/0.6% KI solution. The formation of clear halos around the colonies confirmed amylase expression [49].

### Southern blot analysis

To purify the pSNJ2Int^Mut^ plasmid, a single transformant of the J7-3-F/pSNJ2Int^Mut^ strain was cultured in liquid medium. Due to the naturally low biomass of haloarchaea, ~100 ml of the culture was harvested per sample. The plasmid was then isolated using a plasmid miniprep kit (Tiangen, Beijing, China). The DNA preparations were electrophoresed on two 0.8% agarose gels at 30V for 3h and soaked in either denaturing (0.4 M NaOH and 1 M NaCl) or nondenaturing buffer (20× SSC: 3 M NaCl and 0.3 M trisodium citrate dihydrate, adjusted to pH 7.0 with HCl). The DNA was transferred onto a Hybond-N+ nylon membrane (Cytiva) using the downward capillary transfer method for 16 h at room temperature. After transfer, the membrane was briefly rinsed, and the DNA was firmly cross-linked to the membrane by baking in an oven at 80°C for 2 h. The primers SNJ2-TZ-F and SNJ2-TZ-R were designed to synthesize a probe corresponding to the region from *orf19* to *orf21* of the plasmid (Supplementary Table S9). The probe was labeled using the DIG High Prime DNA Labeling and Detection Starter Kit I (Roche). Pre-hybridization and hybridization were performed using the DIG Easy Hyb buffer (Roche). The DIG-labeled probe was denatured in a boiling water bath for 10 min, immediately chilled on ice, and then added to the pre-warmed buffer. Hybridization was carried out at a strict temperature of 42°C for 4 h to overnight. Post-hybridization washes included two low-stringency washes (2× SSC, 0.1% SDS) at room temperature for 15 min each, followed by two high-stringency washes (0.5× SSC, 0.1% SDS) at 65°C–68°C for 15 min each. Signal detection was performed strictly at room temperature using the anti-digoxigenin-AP conjugate and the NBT/BCIP colorimetric substrate system provided in the aforementioned Roche kit.

### DNA multiple sequence alignment and secondary structure prediction

Multiple sequence alignment of the target DNA sequences (such as the region downstream of *orf19* among *Betapleolipovirus* members) was performed using the MUSCLE algorithm implemented in MEGA11 software with default parameters [50]. The resulting alignments were subsequently visualized and shaded for conservation using GeneDoc [51].

For the thermodynamic prediction of ssDNA secondary structures, the sequences were analyzed using the Mfold web server with default folding parameters [52]. The raw predicted structures generated by Mfold were then used as reliable templates to manually generate the schematic representations for enhanced visual clarity in the figures.

### Protein clustering and analysis

#### Data acquisition and curation

A total of 527 protein sequences annotated with the PF25227 domain were retrieved from the InterPro database (Supplementary Table S5) [53]. This dataset was supplemented with SNJ2 ORF19 homologs identified by PSI-BLAST searches queried with the SNJ2 ORF19 sequence against the nonredundant NCBI protein sequence database. For poorly represented sequences, such as MCC6055023 (*Thermosphaera* sp.), additional searches were performed using HMMER with default parameters against the MGnify database of environmental sequences [54].

#### Sequence filtering and phylogenetic analysis

Short or incomplete sequences that could not be aligned to the core conserved region were excluded. For phylogenetic analysis, the sequence dataset was filtered to 80% identity over 80% of protein length using MMseqs2 [55], yielding a final dataset of 198 sequences. The sequences were aligned using MAFFT v7 [56] with the G-INS-1 option. Noninformative sites were removed using trimal with the gappyout option [57]. Maximum likelihood phylogenetic analysis was performed using IQ-Tree v2.0.6 [58], with the best selected amino acid substitution model being LG + F + R8. The branch support was assessed using SH-aLRT [59].

#### Redundancy reduction and structural candidate selection

To minimize computational load for subsequent structure-based analyses, the combined dataset was subjected to redundancy removal using CD-HIT [60] with strict thresholds of 90% sequence identity and 90% sequence coverage. Only proteins with high-confidence AlphaFold-predicted structures (average pLDDT >70) [61] were retained. While the majority of structures were retrieved from the AlphaFold Protein Structure Database (AlphaFold2), the structures for a small subset of proteins not deposited in UniProt were generated de novo using AlphaFold3. From this curated set, 78 representative proteins were selected to evenly cover the major clades identified in the full-sequence phylogenetic tree.

#### Structure-based comparative analysis

##### Pairwise structural similarity analysis

The conserved domain region of each representative protein was extracted from its AlphaFold-predicted structure based on Pfam-defined boundaries (Supplementary Table S6). Pairwise structural alignments were performed using the TM-score algorithm [62], and the resulting TM-scores were compiled into a structural similarity matrix [62].

##### Structure-based clustering using structural alphabets

To complement the TM-score analysis, the same protein domains were converted into their corresponding 3Di sequences using Foldseek [63]. Multiple sequence alignment was then generated from these 3Di sequences using ClustalW [64]. To optimize the alignment for the structural alphabet, we used an identity matrix for substitutions and set the gap extension penalty to 0.1. Finally, based on the obtained 3Di sequence alignment, a structural dendrogram was generated using the UPGMA (Unweighted Pair Group Method with Arithmetic Mean) algorithm with 1500 bootstrap replicates on MEGA [65].

#### Data visualization

The structural similarity matrix was visualized using Morpheus (https://software.broadinstitute.org/morpheus). The structural dendrogram generated from the UPGMA clustering was visualized and annotated using iTOL (interactive Tree Of Life) web server [66]. High-quality molecular graphics of the protein 3D structures were rendered using UCSF Chimera [67].

## Results

### The integrase coding sequence is critical for SNJ2 genome replication

The genomes of betapleolipoviruses typically exists as circular double-stranded DNA molecules both within virions and during intracellular replication. Notably, characteristic single-strand interruptions have been detected in the virion-packaged genomes of several members, including SNJ2 and HRPV3 [41, 68]. Despite these interruptions, the genomes remain overall circular double-stranded DNA molecules, theoretically making them ideal backbones for constructing shuttle plasmids (Supplementary Fig. S1). However, to date, no genetic manipulation system based on this group of viruses has been reported. In an effort to construct a *Natrinema*-*E. coli* shuttle vector using SNJ2 as a backbone, we hypothesized that deleting the integrase gene from the SNJ2 genome would yield a plasmid capable of stable replication in host cells. This assumption was based on the prevailing view that integrases in temperate viruses function solely in mediating viral genome integration into or excision from the host chromosome and are not directly involved in viral genome replication. For example, it has been previously demonstrated that the complete deletion of the integrase gene in the fusellovirus SSV1 did not impair its stable replication and infection [69].

**Figure 1.**
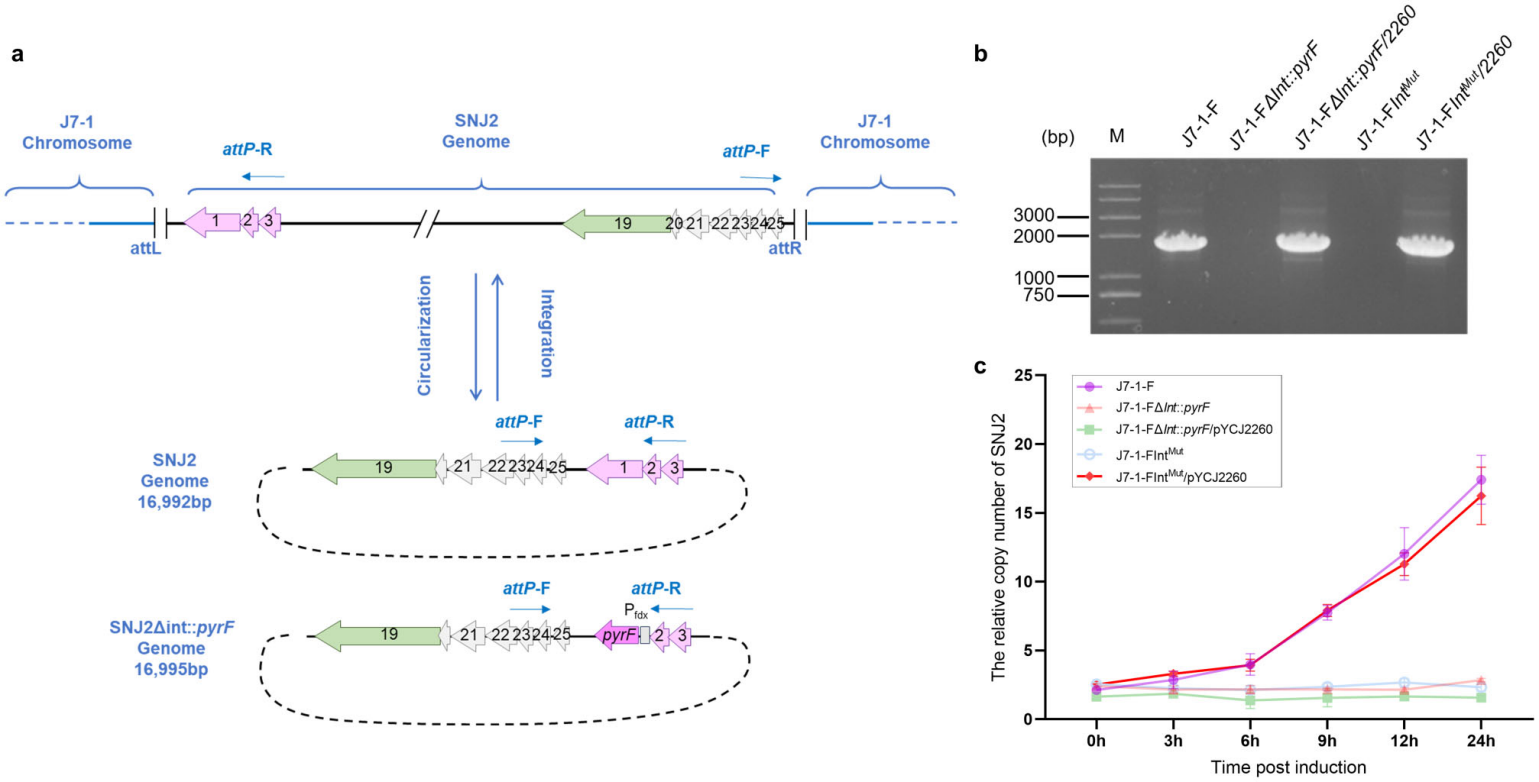
The integrase coding sequence is critical for SNJ2 genomic replication. (**a**) The upper panel illustrates the gene arrangement of SNJ2 in its integrated state, with the host genome shown and dashed lines indicating omitted segments of the genome. The lower panel shows the arrangement of the viral genome following excision and circularization (SNJ2 genome and SNJ2Δint::*pyrF*). Arrows represent ORFs and their transcriptional orientation. The primer pair (*attP* F/R) used for *attP*/*attP*′ detection is indicated in the schematic, yielding PCR products of 1859 bp for *attP* from the SNJ2 genome template and 1862 bp for *attP*′ from the SNJ2Δint::*pyrF* template. The *attL* and *attR* junctions flanking the integrated provirus, as well as the *attP* junction generated upon excision, are indicated at the corresponding boundaries. The *pyrF* cassette inserted at the int locus is indicated. (**b**) Detection of the viral circularization junction *attP*/*attP*′ by standard PCR after MMC induction using the primer pair *attP* F/R described in panel (a). J7-1-F (wild type), J7-1-FΔint::*pyrF* (*int* replaced with *pyrF*), J7-1-FΔint::*pyrF*/pYCJ2260 (*int* replaced with *pyrF* and complemented with *int* from a plasmid), J7-1-FInt^Mut^ (*int* was prematurely terminated with stop codons), and J7-1-FInt^Mut^/pYCJ2260 (*int* was prematurely terminated with stop codons and complemented with *int* from a plasmid). (**c**) The strains in panel (b) were cultured to mid-exponential phase (OD_600_ = 0.6–0.8) in Halo-2 medium. Cells were treated with MMC (1 µg/ml) for 30 min, then harvested by centrifugation, washed twice with Halo-2 medium to remove residual MMC, and resuspended in fresh Halo-2 medium. Samples were collected every three hours post-induction, and the relative copy number of the SNJ2 genome was quantified by qPCR using primer pairs *orf*7-F/*orf*7-R (for the viral genome) and *radA*-F/*radA*-R (for the host chromosome). The experiment was performed using three independent biological replicates, with error bars representing the standard deviations of the biological replicates.

To test this hypothesis, we constructed an integrase gene deletion mutant in strain J7-1-F, designated J7-1-FΔ*int::pyrF*, via homologous recombination (Fig. 1a). As expected, treatment of this mutant with MMC failed to generate detectable circularized viral genome junctions (*attP*), confirming the loss of integrase activity. Upon complementation with plasmid pYCJ2260, which carries the complete integrase gene operon (*orf1-3* of SNJ2), the *attP* junctions were once again detectable (Fig. 1b). Surprisingly, however, the copy number of the circularized SNJ2 genome lacking the integrase gene did not increase over time (Fig. 1c), indicating a loss of replication ability.

To investigate the role of the integrase coding sequence in SNJ2 replication, we introduced two premature stop codons at amino acid positions Arg88 and Gln90 of the integrase gene to ensure complete translational termination, thereby creating the mutant strain J7-1-F SNJ2Int^Mut^. Similar to the deletion mutant, this strain showed no *attP* junctions upon MMC induction, confirming that the nonsense mutations abolished integrase function. Nevertheless, when complemented with pYCJ2260, the mutant SNJ2 genome recovered replication ability comparable to that of the wild-type virus (Fig. 1c). These results indicate that the presence of the integrase coding sequence, rather than its catalytic activity, is essential for SNJ2 genome replication. We therefore conclude that the integrase gene performs a critical and previously unrecognized role in supporting SNJ2 replication, beyond its canonical function in genome integration/excision.

### Excision and circularization of the SNJ2 provirus enables its terminal regions to form a novel, functional integrase-containing operon that defines the viral replication region

In the circularized SNJ2 genome, the integrase gene (*orf1*) and its upstream neighboring genes (*orf2* and *orf3*) [40], which are co-transcribed in the proviral state, join with seven additional ORFs from the right terminus of the proviral genome (*orf19-25*) to form an extended operon-like structure (Supplementary Fig. S2a). In addition, circularization generates a novel coding sequence, designated *orf26*, which includes the *attP* junction and is located downstream of the integrase gene [70]. Expression of ORF26 was confirmed by GFP-tagging and western blotting (Supplementary Fig. S2b), demonstrating that this newly formed ORF indeed encodes a stable protein capable of being translated *in vivo*. Altogether, the putative operon comprises 11 compactly arranged ORFs, with adjacent genes exhibiting minimal gaps or short overlaps (detailed in Supplementary Table S1). Such compact genomic organization, reminiscent of bacterial operons, suggests that these ORFs may be co-transcribed as a functional unit in the circularized genome (Supplementary Fig. S2a). Transcriptional analysis confirmed that *orf19* through *orf1* are co-transcribed as a single transcriptional unit (Supplementary Fig. S2c). Importantly, the transcription start site was mapped within the integrase gene (*orf1*), 419 nucleotides upstream of its stop codon (adenine residue), while the termination site was identified 50 nucleotides downstream of *orf19*, thereby delineating the precise boundaries of this extended operon (Supplementary Fig. S2a).

Given our finding that the integrase coding sequence is essential for SNJ2 replication, we hypothesized that this novel operon may play a critical role in viral replication. To test this hypothesis, we systematically introduced precise nonsense mutations into *orf19* through *orf26* in the chromosome of strain J7-1-F, thereby abolishing the expression of each respective gene product. Following MMC induction, which induces the SNJ2 excision [49], *attP* junctions were detected in all mutant strains, indicating that viral excision and circularization remained unaffected (Supplementary Fig. S2d). However, none of the circularized SNJ2 genomes from these mutants exhibited increased copy numbers, resembling the phenotype of the strain in which the integrase gene was provided in trans. As a control, the structural gene deletion mutant (Δ*orf13*) exhibited a genome replication phenotype identical to that of the wild-type strain (Supplementary Fig. S2e). These results demonstrate that translation of each ORF into a functional protein in this locus is essential for SNJ2 genome replication. Function of each of these ORFs were predicted using HHpred and provided in Supplementary Table S2.

**Figure 2.**
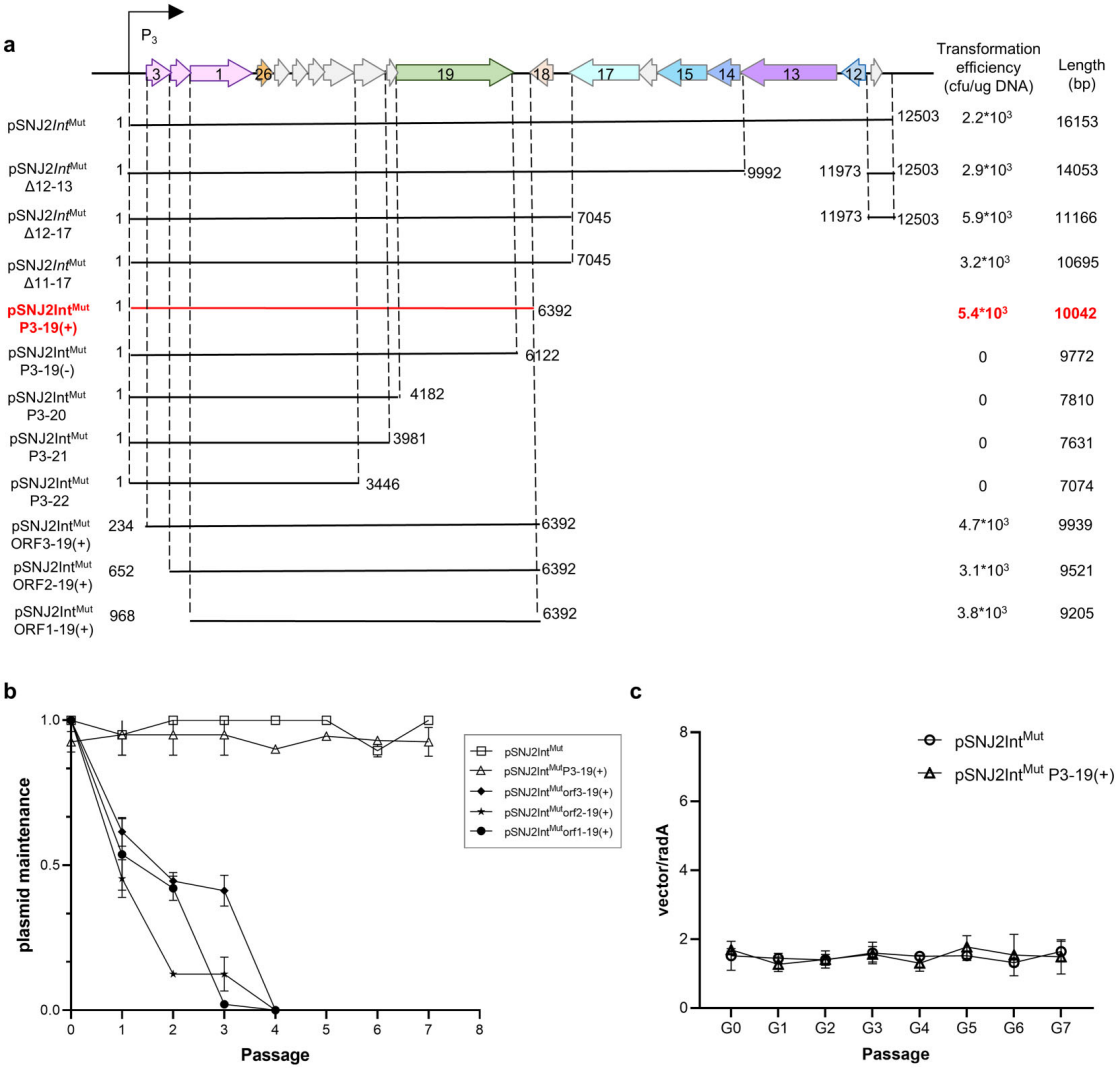
A new operon harboring the integrase gene emerges after SNJ2 genome circularization. (**a**) Schematic representations of pSNJ2Int^Mut^ vectors containing various deletions of the full-length SNJ2, excluding regulatory regions, are shown along with their corresponding transformation efficiencies. Horizontal arrows represent ORFs, with numbers indicating their identities and directions of transcription. Solid lines represent different fragments, with numbers denoting start and end positions. The minimal replicon of SNJ2 is highlighted. The (+) and (−) symbols indicate the presence or absence, respectively, of the noncoding DNA region downstream of *orf19*. (**b**) Stability and maintenance of the shuttle vectors. Shuttle vectors were maintained in J7-3-F cultures supplemented with uracil. J7-3-F cultures (50 μl) were diluted in 5 ml of Halo-2 medium and incubated for 24 h. After each of the seven serial dilutions, aliquots were spread on Halo-2 medium, and 50 random colonies were selected and spotted onto 20% MM plates without uracil. Maintenance was assessed by calculating the percentage of colonies capable of complementation. The results represent the averages of three independent experiments, with error bars indicating standard deviations. The pSNJ2Int^Mut^ plasmid exhibited 100% maintenance across all independent biological replicates during multiple passages. Therefore, the standard deviation is zero and no error bars are plotted for this group. (**c**) Relative copy number of pSNJ2Int^Mut^ and pSNJ2Int^Mut^ P3-19(+) plasmids in J7-3-F. The relative plasmid copy number (vector/*radA*) was determined by qPCR following successive subculturing under nonselective (antibiotic-free) conditions. Data are presented as the mean ± SD from three independent biological replicates.

To define the minimal replicon of SNJ2, encompassing all essential ORFs required for autonomous replication and stable partitioning, we constructed a plasmid in which the previously validated lysogeny/lysis regulatory region (*orf4*–*orf10*) was deleted [49]. The remaining SNJ2 genomic fragment (*orf3*–*orf11*, 12 503 bp) was inserted to replace the halophilic replication element in the *Natrinema-E. coli* shuttle vector pFJ6 [71], yielding a new plasmid designated pSNJ2Int^Mut^ (Supplementary Fig. S3a). The two nonsense mutations in the integrase gene described earlier were introduced via high-fidelity PCR using a pair of mutagenic primers (details in supplementary material; Fig. 2a).

Building upon pSNJ2Int^Mut^, we generated a series of truncation derivatives of the SNJ2 genomic insert (Fig. 2a). As expected, the SNJ2 replication region was precisely mapped to the locus corresponding to the novel operon formed following proviral excision and circularization. Notably, partial truncation of the region upstream of the integrase gene (*orf1*) still allowed transformation, but the resulting plasmids exhibited significantly reduced stability (Fig. 2b). In contrast, complete deletion of the region downstream of *orf19* completely abolished plasmid transformation. These results indicate that both the upstream and downstream flanking regions of this operon are essential for efficient replication of SNJ2, prompting a more detailed investigation of this sequence element. Through this systematic mapping, we identified pSNJ2Int^Mut^P3-19(+) as the minimal segment encompassing all necessary components for robust replication and stable maintenance (Fig. 2b). The extrachromosomal, free-replicating state of both pSNJ2Int^Mut^ and pSNJ2Int^Mut^P3-19(+) was supported by *attP*/*attL* PCR analysis and successful plasmid rescue experiments (Supplementary Fig. S4a and b). Furthermore, quantification of their relative abundance showed that both plasmids were maintained at similar copy numbers, ~1 copy per chromosome (Fig. 2c). Given the highly polyploid nature of haloarchaea, a plasmid-to-chromosome ratio of ~1 corresponds to multiple episomal copies per cell and is consistent with stable maintenance under uninduced conditions [72–74].

Leveraging the autonomy of the SNJ2 replication region, we further developed a shuttle vector to complement existing genetic tools for *Natrinema* sp. J7 (Supplementary Fig. S3b). Functional characterization revealed that the SNJ2-based vector is stably maintained and fully compatible with previously established SNJ1-based (pYC-SHS) and chromosome-derived (pFJ6) plasmids, allowing for complex co-transformation setups (Supplementary Fig. S4c and d). While its host range appears largely restricted to the genus *Natrinema* (e.g. *N. gari*), the vector proved highly effective for heterologous gene expression, successfully driving the production of active amylase (Supplementary Fig. S4e) and functional viral immunity elements (Supplementary Fig. S4f). A detailed description of vector construction, stability assays, host range analysis, and expression tests (e.g. amylase activity) is provided in the supplementary text, Supplementary Figs S3 and S4. The successful coexistence of this vector with SNJ1-derived plasmids offers a valuable approach for dual-gene expression and future mechanistic studies on virus–virus interactions [31].

### The sequence downstream of SNJ2-ORF19 is conserved and essential in betapleolipoviruses

**Figure 3.**
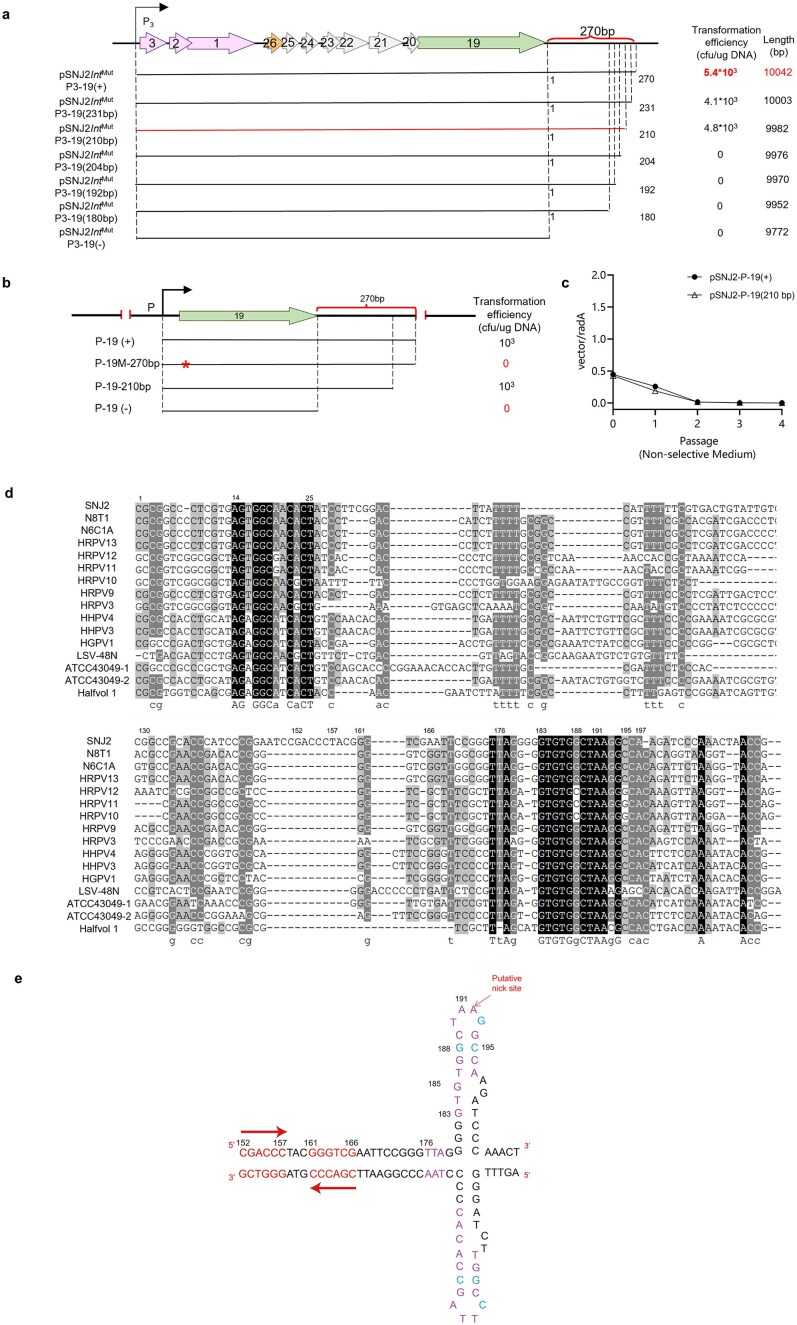
The sequence downstream of *orf19* is essential for viral replication and widely conserved among *betapleolipoviruses*. (**a**) Stepwise truncation of the *orf19* downstream sequence defines the region essential for SNJ2 replication. The transformation efficiencies of the constructs are presented on the right. pSNJ2^Mut^P3-19(210bp) is the shortest construct capable of yielding transformants and includes a 210-nt downstream region of *orf19*. (**b**) Analysis of the importance of ORF19 for replication. Horizontal arrows indicate the position of ORF19. Transformation efficiencies of the corresponding constructs are shown on the right. The 19M mutant carries a premature stop codon introduced within *orf19*, indicated by an asterisk (*). The (+) and (−) symbols indicate the presence and absence of the downstream noncoding DNA region of ORF18-19. (**c**) Relative copy numbers of shuttle vectors in J7-3-F respectively in complete medium. Results are averages for three independent experiments, and error bars indicate standard deviations. (**d**) Multiple sequence alignment of the region downstream of *orf19* across members of the genus *Betapleolipovirus*. Sequences were aligned using MEGA software and visualized with GeneDoc. Representative sequence alignments shown include *Haloarcula marismortui* ATCC 43049 and *Haloferax volcanii* DS2 (Halfvol1 provirus). Full genomic details are provided in Supplementary Table 3. (**e**) Predicted secondary structure of the ORF19 downstream region (152–209 nt). The schematic was drawn based on the thermodynamic prediction of the ssDNA sequence using the Mfold web server [52]. The raw prediction output from Mfold is provided in Supplementary Fig. S5. The predicted structure is color-coded based on sequence conservation: completely conserved bases are shown in purple and highly conserved bases are in blue. The predicted stem–loop structure formed by the upstream IR (152–166 nt) is highlighted in red. Other less conserved regions that do not form secondary structures are shown in black.

To delineate the minimal DNA segment required for plasmid replication downstream of *orf19*, we performed stepwise truncations of the 270-bp intergenic sequence between *orf19* and *orf18* based on the pSNJ2Int^Mut^P3-19(+) construct. As shown in Fig. 3a, replication was sustained by a 210-bp fragment, while further deletions abolished transformation efficiency entirely.

As described above, the circularized operon formed upon SNJ2 excision constitutes the replication module of the viral genome. Within this region, *orf19* is the only gene conserved across members of the *Betapleolipovirus* genus (Supplementary Fig. S1), suggesting a key role in replication. To test this, we replaced the native replication region of the haloarchaeal plasmid pFJ6 with *orf19* and its 210-bp downstream sequence (Fig. 3b). The resulting construct pSNJ2-P-ORF19(+) (expressing ORF19 under a strong promoter) was capable of autonomous replication and yielded transformants on selective plates. Consistent with the polyploid nature of haloarchaea, an initial plasmid-to-chromosome ratio of ~0.5 corresponds to the successful establishment of multiple plasmid copies per cell. To verify the physical state of the plasmid, we performed rescue experiments that confirmed that pSNJ2-P-ORF19(+) is maintained as an extrachromosomal episome (Supplementary Fig. S4b). However, the plasmid was frequently lost during subsequent passages, indicating instability in host cells (Fig. 3c). In contrast, either a nonsense mutation in *orf19* (pSNJ2-P-ORF19M) or truncation of the critical downstream sequence [pSNJ2-P-ORF19(−)] completely abolished transformation, confirming that both the functional ORF19 protein and its adjacent downstream region are indispensable for replication (Fig. 3b).

Although unstable, the pSNJ2-P-ORF19(+) construct provided a platform for functional characterization of the 210-bp region. Sequence alignments revealed that this region contains two highly conserved motifs shared among members of the *Betapleolipovirus* genus (Fig. 3d). The first motif (positions 1–36) lies near the transcription termination site of the replication operon, suggesting its role in transcription termination. The second conserved motif (positions 176–197) is located at the distal end and coincides with the essential replication element defined by our truncation analysis. Secondary-structure prediction using Mfold indicated a conserved stem–loop (hairpin) structure within this distal segment (Fig. 3e and Supplementary Fig. S5) [52]. Furthermore, we identified an additional inverted repeat (IR) sequence (156–166 nt) located upstream of the conserved hairpin (Fig. 3e). Further sequence analysis revealed that the presence of an IR upstream of the conserved stem–loop structure is a common feature across the *Betapleolipovirus* genus, although its sequence and precise spacing from the distal hairpin are not conserved.

This bipartite architecture is characteristic of canonical RCR origins, such as that of the pT181 plasmid family [7, 9, 75, 76]. In these bacterial models, the replication initiator protein (Rep) binds to a double-stranded IR (the bind site) and subsequently nicks a conserved stem–loop (the nick site) in its single-stranded loop. This led us to hypothesize that the identified upstream IR (156–166 nt) and the distal hairpin (176–197 nt) function as the cognate bind and nick sites for ORF19, respectively.

To experimentally validate the importance of the secondary structure elements of this region, we used pSNJ2-P-ORF19(+) as a backbone for site-directed mutagenesis. First, we targeted the conserved distal hairpin (176–197 nt). As shown in Supplementary Fig. S6A and Table 1, mutations designed to disrupt stem base-pairing (M3, M4) or mutate the loop (M2) completely abolished replication. In contrast, a mutation (M1) that preserved the hairpin structure had no effect, confirming that its structural integrity is essential. Second, we introduced three distinct mutations into the upstream IR (156–166 nt), which functions as the predicted double-stranded bind site (Supplementary Fig. S6b and Table 1). Mutants with a symmetry-preserving mutation M5 and a symmetry-disrupting mutation on the 5′ half-site M6 both remained fully functional, exhibiting wild-type transformation efficiencies. In striking contrast, a symmetry-disrupting mutation on the 3′ half-site (M7) severely impaired replication, resulting in a 100-fold reduction in transformation efficiency. Thus, the pSNJ2-P-ORF19(+) *ori* is a complex bipartite element, requiring both a highly conserved distal hairpin and a nonconserved upstream bind site for full activity.

**Table 1.** Effect of mutations in the ORF19 downstream region on plasmid replication

Mutant	Target element	Mutation (site and change)	Predicted structural effect	Replication (±)	Transformation efficiency (cfu/ng DNA)
Wide type	−	−	−	+	1.9*10^3^
M1	Stem	G188CC195G	Structure-preserving	+	2.0*10^3^
M2	loop	A191TA192T	Loop-mutation	−	0
M3	Stem	T186GG187T	Stem-disrupt	−	0
M4	Stem	C195AC196A	Stem-disrupt	−	0
M5	Invert repeat sequence	C156GC157GG161CG162C	Symmetry-preserving (sequence altered)	+	1.7*10^3^
M6	Invert repeat sequence	C156TC157T	Symmetry-disrupting(5′ half-site)	+	2.1*10^3^
M7	Invert repeat sequence	G161AG162AG163AT164C	Symmetry-disrupting(3′ half-site)	+	5.0*10^1^

Given the strong conservation of the distal element within the *Betapleolipovirus* genus, we examined its distribution across related archaeal genomes. BLASTn searches using the conserved DNA motif (183–197 nt) as a query revealed its wide spread across numerous halophilic archaeal genomes. In the *Haloarcula marismortui* ATCC 43049 chromosome, two highly similar sequences were identified (Supplementary Table S3): one located within an integrated element and another within a predicted *Betapleolipovirus* proviral region. Both motifs are positioned immediately downstream of an ORF19-like gene (Fig. 3d and Supplementary Table S3). A similar pattern was observed in the *Haloferax volcanii* DS2 genome, where one match was found within the previously reported Halfvol1 provirus, also adjacent to an ORF19-like gene (Fig. 3d and Supplementary Table S3). These observations strongly suggest a functional interdependence between the replication-associated ORF19-like protein and its downstream conserved DNA motif, further supporting the conclusion that their co-occurrence is essential for proper biological activity.

### ORF19 functions as a novel replication initiator with structural homology to the Rep_trans family

**Figure 4.**
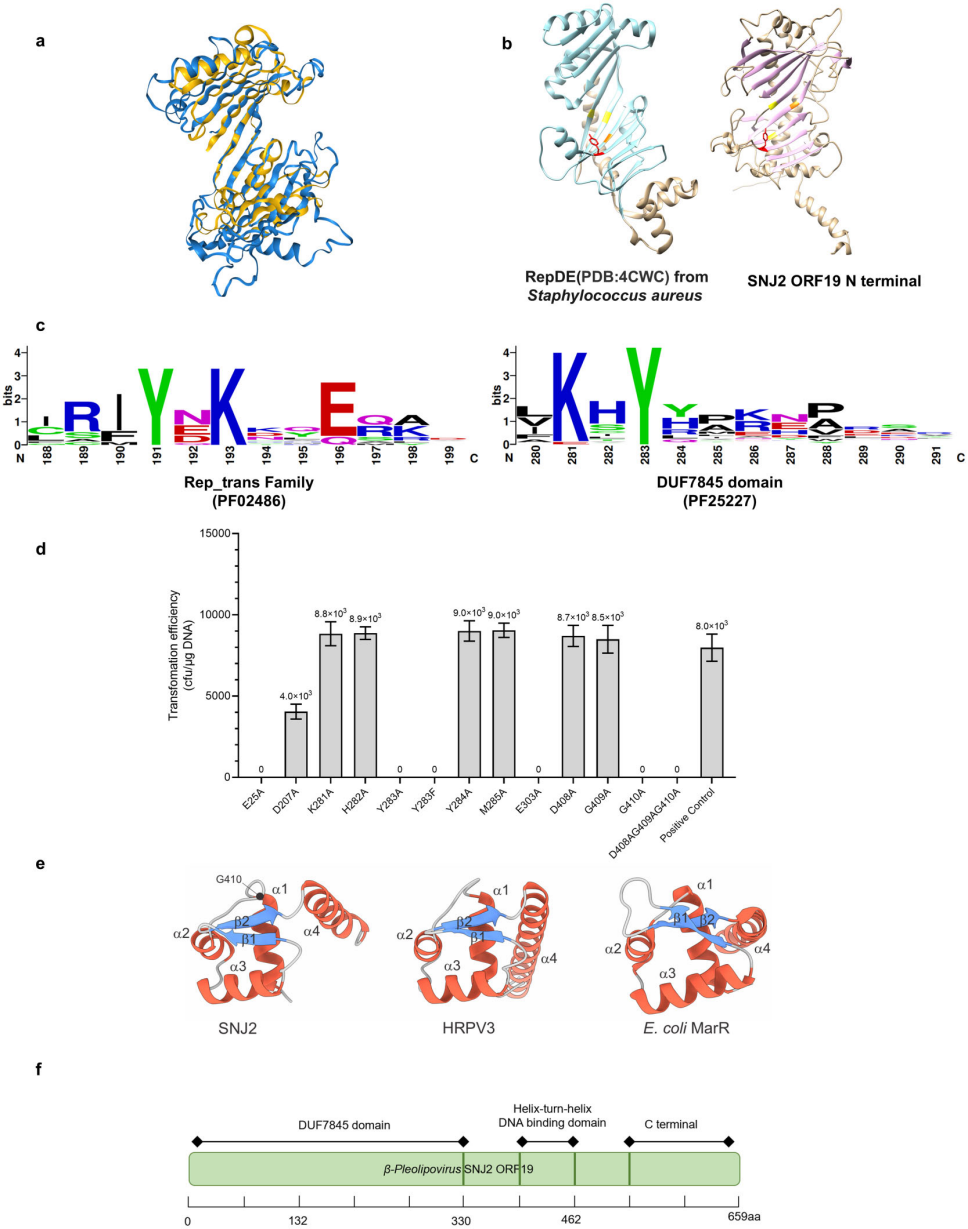
ORF19 (RepSNJ2) and RepDE share a common active catalytic site. (**a**) Structural conservation between AlphaFold-predicted ORF19 (RepSNJ2) and a putative replication protein from *Inoviridae* sp. The 3D structural superposition reveals significant similarity, particularly within the N-terminal domain, between the AlphaFold-predicted ORF19 (RepSNJ2) (blue) and the *Inoviridae* protein A0A345MNV4 (gold). While the structural similarity was initially identified by Foldseek, the quantitative structural alignment performed using TM-align yielded an RMSD of 4.67 Å and a TM-score of 0.54, confirming a highly conserved global topological fold. (**b**) Crystal structure of RepDE1 (PDB ID: 4CWC) and the AlphaFold-predicted ORF19 (RepSNJ2) structure. Quantitative structural alignment of the core domains using TM-align yielded an RMSD of 4.47 Å and a TM-score of 0.49. The conserved catalytic tyrosine (Y191) and the three metal-coordinating residues (D46, D142, and E214) essential for its activity are colored (Y, red; D, yellow; E, orange). The corresponding conserved residues in the ORF19 (RepSNJ2) structure are labeled with the identical color scheme, demonstrating the precise spatial conservation of the catalytic center. (**c**) WebLogo representations of the conserved Motif III of Rep_trans protein family (consensus: YxxK/YxxKY) and DUF7845 containing homologs. The left panel shows the motif in the Rep_trans protein family, and the right panel shows the corresponding motif in DUF7845 containing homologs. Amino acid residues are colored by chemical property. (**d**) Functional analysis of ORF19 (RepSNJ2) active-site mutants. Transformation efficiencies in J7-3-F were measured for plasmids expressing ORF19 (RepSNJ2) variants. Mutations targeted key predicted functional sites: (i) the catalytic motif, including the essential tyrosine (Y283A and Y283F) and surrounding conserved residues (K281A, H282A, Y284A, M285A); (ii) conserved acidic residues predicted for metal ion coordination (E25A, D207A, E303A); (iii) conserved residues within the C-terminal DNA-binding domain (D408A, G409A, G410A). Data represent the mean of three biological replicates, with error bars indicating standard deviation. (**e**) Structural superposition of the putative HTH d7omains of SNJ2 ORF19 (accession: YP_010 772 542, truncated, 395–469, left) and the HHPV3 ORF19-like protein ORF11 (accession: YP_009 798 574, truncated, 376–466, center) with MarR family regulator from *E. coli* (PDB: 1JGS, truncated, 38–125, right) [78]. (**f**) Schematic of the domain organization of ORF19 (RepSNJ2). Positions of conserved motifs are noted.

To explore the molecular basis of the ORF19 activity, we performed structural homology searches queried with the AlphaFold 3-predicted model of ORF19 using Foldseek [77]. Searches against the PDB database yielded only marginally significant (E > 2.6–2) hits covering the ~80 aa-long central region of ORF19, which corresponds to the wHTH domain (see below). However, when the search was performed against the database of viral protein structures, hits were also obtained for the N-terminal region (~300 aa), which is classified as a DUF7845 domain (PF25227) of currently unknown function. The hits were to various TBP-fold proteins, including TBP encoded by large eukaryotic viruses (e.g. A0A2N9QVX7, Dishui lake phycodnavirus 1, E = 5.7e-5, probability 1.0), but also to the replication initiator from *Inoviridae* sp. (A0A345MNV4_9VIRU, E = 2.76e-3, probability 0.97) (Fig. 4a) which contains the PF02486 (Rep_trans) domain. Structural alignment of ORF19 with the crystal structure of RepDE from *Staphylococcus aureus* (PDB ID: 4CWC), an experimentally characterized representative of the Rep_trans family [7], revealed an overlap with the catalytic domain of the latter protein (Fig. 4b). Importantly, structural superposition of the two proteins showed that ORF19 contains a tyrosine residue at the position occupied by the catalytic tyrosine of RepDE [7] (Fig. 4b), suggesting that ORF19 also functions as the initiator of the RCR.

Multiple sequence alignment of DUF7845-containing homologs (provided in Supplementary Dataset S1) showed that, despite low overall sequence similarity, the putative catalytic tyrosine residue is absolutely conserved, as illustrated by the WebLogo analysis (Fig. 4c). In canonical members of the Rep_trans family, the catalytic tyrosine which mediates DNA cleavage and covalent linkage is highly conserved and is embedded within a conserved context, YxxK/YxxKY, known as Motif III (Fig. 4c) [7, 62–64]. By contrast, the sequence surrounding the catalytic tyrosine in SNJ2 ORF19 forms a distinct KHYYM motif (residues 281–285). While this sequence diverges from the canonical consensus, alignment of SNJ2-like pleolipoviruses reveals that the KHYYM motif is highly conserved within this specific lineage (Supplementary Fig. S7). To investigate the functional relevance of this conserved motif, we performed alanine scanning mutagenesis (K281A, H282A, Y283A, Y284A, and M285A). Strikingly, the results showed that only Y283 was critical for activity, whereas mutations of other residues had no effect on plasmid replication (Fig. 4d). Furthermore, a conservative phenylalanine substitution (Y283F) exhibited a lethal phenotype identical to that of Y283A (Fig. 4d). These results pinpoint Y283 as the sole residue within the catalytic motif indispensable for replication initiation, and explicitly demonstrate the strict requirement of the hydroxyl group for the catalytic cleavage.

Besides the catalytic tyrosine, ORF19 contains three acidic residues (E25, D207, E303) at positions equivalent to those of D46, D142, and E214 in RepDE, which were predicted to coordinate a metal ion in the catalytic center [7]. Whereas substitution D207A had minimal effect on replication, the activity was abolished in E25A and E303A mutants (Fig. 4d), supporting their role in active-site stabilization and phosphodiester hydrolysis.

Structural analysis revealed that the N-terminal catalytic DUF7845 domain of SNJ2 ORF19 is followed by a wHTH domain, conserved in other betapleolipoviruses (e.g. HHPV3, YP_009 798 574), and most closely resembling those in MarR family transcriptional regulators from *E. coli* (PDB: IJGS) (Fig. 4e) [78]. To verify the functional importance of the potential DNA-binding region, we attempted to destabilize the wHTH domain by introducing mutations (D408A, G409A, G410A) within the conserved linker between α1 and α2 helices within this region. Whereas D408A and G409A had no effect on the plasmid replication, G410A abolished plasmid replication, suggesting that wHTH domain contributes to DNA binding or structural integrity of the replication complex (Fig. 4d and Supplementary Fig. S7). The central wHTH domain is followed by an additional globular α/β domain which, however, does not yield matches to functionally characterized domains.

Importantly, to rule out the possibility that the observed loss of replication activity was an artifact of protein instability or degradation, we assessed the expression of the wild-type ORF19 and its corresponding mutant variants using a pFJ6-based overexpression system. Western blot analysis confirmed that all of the mutant proteins were successfully expressed and maintained stability comparable to that of the wild-type protein (Supplementary Fig. S8). This biochemical evidence further supports the conclusion that the defective replication phenotypes were caused by the mutations in the conserved sites.

Together, these results demonstrate that ORF19 is a previously uncharacterized replication initiator that combines an N-terminal DUF7845 catalytic domain, which we show here to be distantly related to Rep_trans endonucleases, with a central MarR-like DNA-binding region, and a C-terminal domain of unknown function, defining a novel family of archaeal replication proteins (Fig. 4f). Based on these functional and structural characteristics, we propose to rename ORF19 as RepSNJ2.

### Detection of single-stranded DNA intermediates supports the rolling-circle replication mechanism for SNJ2

**Figure 5.**
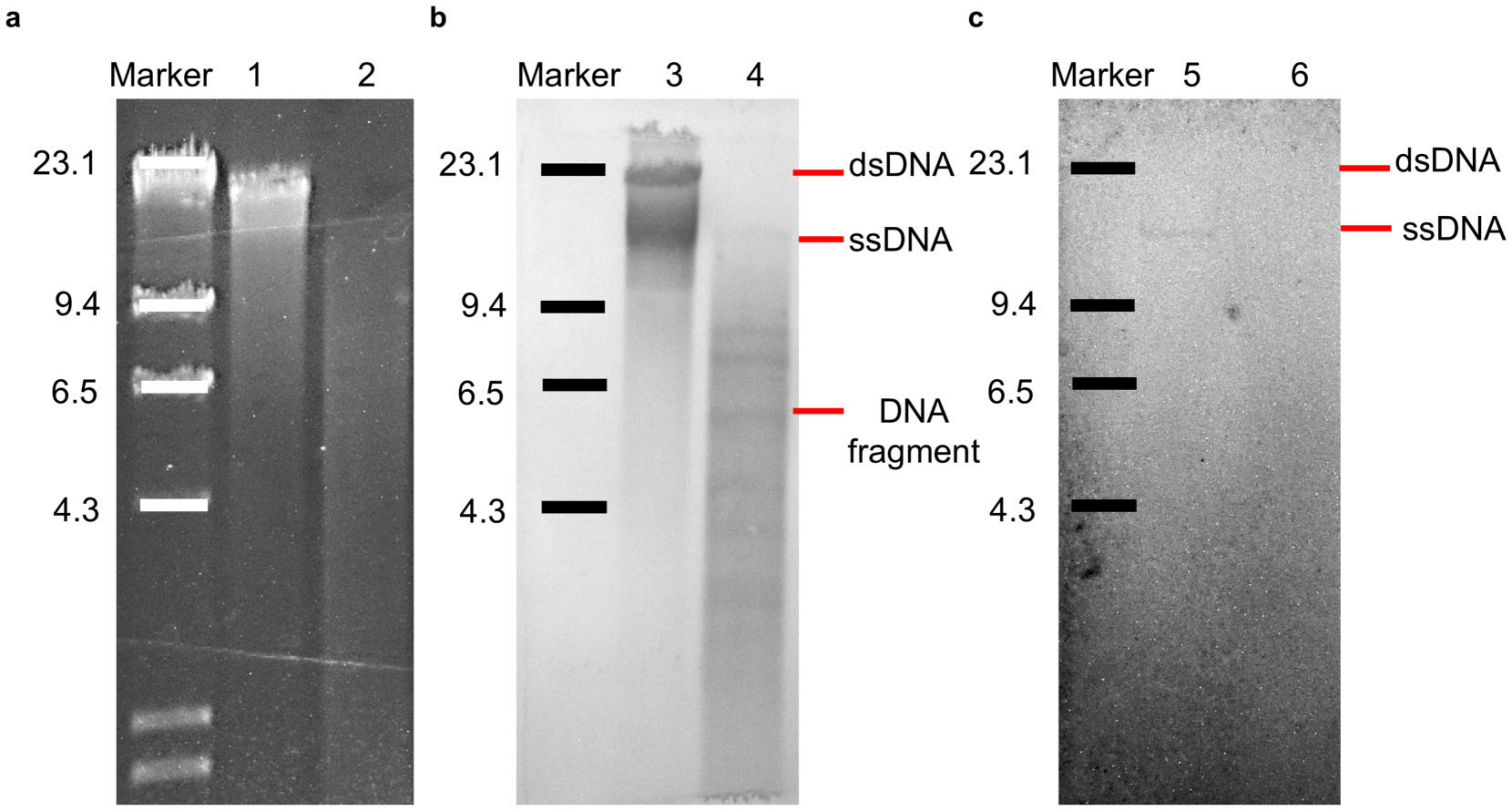
Detection of replication intermediates by Southern blot hybridization. (**a**) Agarose gel electrophoresis of plasmid DNA extracted from J7-3-F/pSNJ2Int^Mut^ transformants. Samples were either untreated (lanes 1, 3, 5) or treated with MBN (lanes 2, 4, 6). (**b, c**) Southern blot analysis of the same samples. After electrophoresis, gels were incubated in either denaturing buffer (b) or nondenaturing buffer (c), followed by transfer to positively charged nylon membranes. Hybridization was performed using DIG-labeled SNJ2-specific probes. MBN digestion selectively removed ssDNA, enabling detection of ssDNA replication intermediates under both denaturing and native conditions.

The occurrence of ssDNA intermediates is a hallmark of the RCR [79, 80]. To experimentally validate that SNJ2 employs this mechanism, we examined whether ssDNA intermediates are indeed generated during the replication. Plasmid DNA was extracted from J7-3-F/pSNJ2Int^Mut^ transformants and subjected to treatment with Mung Bean Nuclease (MBN), an ssDNA-specific nuclease. Both MBN-treated and untreated samples were analyzed by Southern blotting. The obtained DNA fragments were separated by neutral gel electrophoresis (Fig. 5a) and subsequently transferred to a positively charged nylon membrane for hybridization under either alkaline (Fig. 5b) or neutral conditions (Fig. 5c).

Under alkaline denaturing conditions, MBN treatment not only eliminated the free ssDNA signal but also abolished the dsDNA signal. The latter was replaced by a distribution of DNA fragments characterized by several distinct, prominent bands (ranging from ~3 to 8 kb) superimposed on a background smear. This specific fragmentation pattern indicates the presence of highly discrete, MBN-sensitive cleavage sites on the replicating plasmid. While the exact nature of these discontinuous sites requires further experimental validation, we hypothesize that they represent transient, unligated lagging-strand intermediates generated during active RCR, a discontinuous synthesis mechanism well-documented in classical models such as bacteriophage ΦX174 [81, 82]. Importantly, under neutral (nondenaturing) conditions (Fig. 5c), a distinct signal corresponding to the displaced ssDNA intermediate was observed in the untreated sample but was completely absent in the MBN-treated counterpart. This MBN-sensitive signal provides direct evidence for the accumulation of ssDNA intermediates during SNJ2 replication. Taken together, these findings strongly support the conclusion that SNJ2 replicates via rolling-circle mechanism.

### Broad distribution of the novel Rep-Arvir family

Having functionally validated that ORF19 is the replication initiator of RepSNJ2, we explored the distribution of its homologs in archaea and their MGEs. To this end, we extracted all members of the Pfam family PF25227, currently annotated as a domain of unknown function (DUF7845), from the InterPro database and supplemented this dataset with homologs identified through PSI-BLAST searches against the nonredundant protein database. DUF7845-domain proteins were found to be exclusive to archaea, widely distributed in members of the phylum *Methanobacteriota*, particularly in extremely halophilic archaea of the class *Halobacteria*, including the basal lineage *Afararchaeaceae* (MDY6774844.1) [83], but also present in methanogenic archaea of the classes *Methanomicrobia* and *Candidatus* Methanofastidiosia, and hyperthermophilic archaea of the class *Thermococci*. Notably, we also retrieved homologs affiliated to archaea of the phyla *Thermoproteota* and *Thermoplasmatota*, and a single homolog was identified in the phylum *Promethearchaeota* (Asgard archaea). Whether the latter sequence genuinely originates from an Asgard archaeon (or rather its MGE) remains to be verified once more sequences of Asgard MGEs become available (Supplementary Table S4).

**Figure 6.**
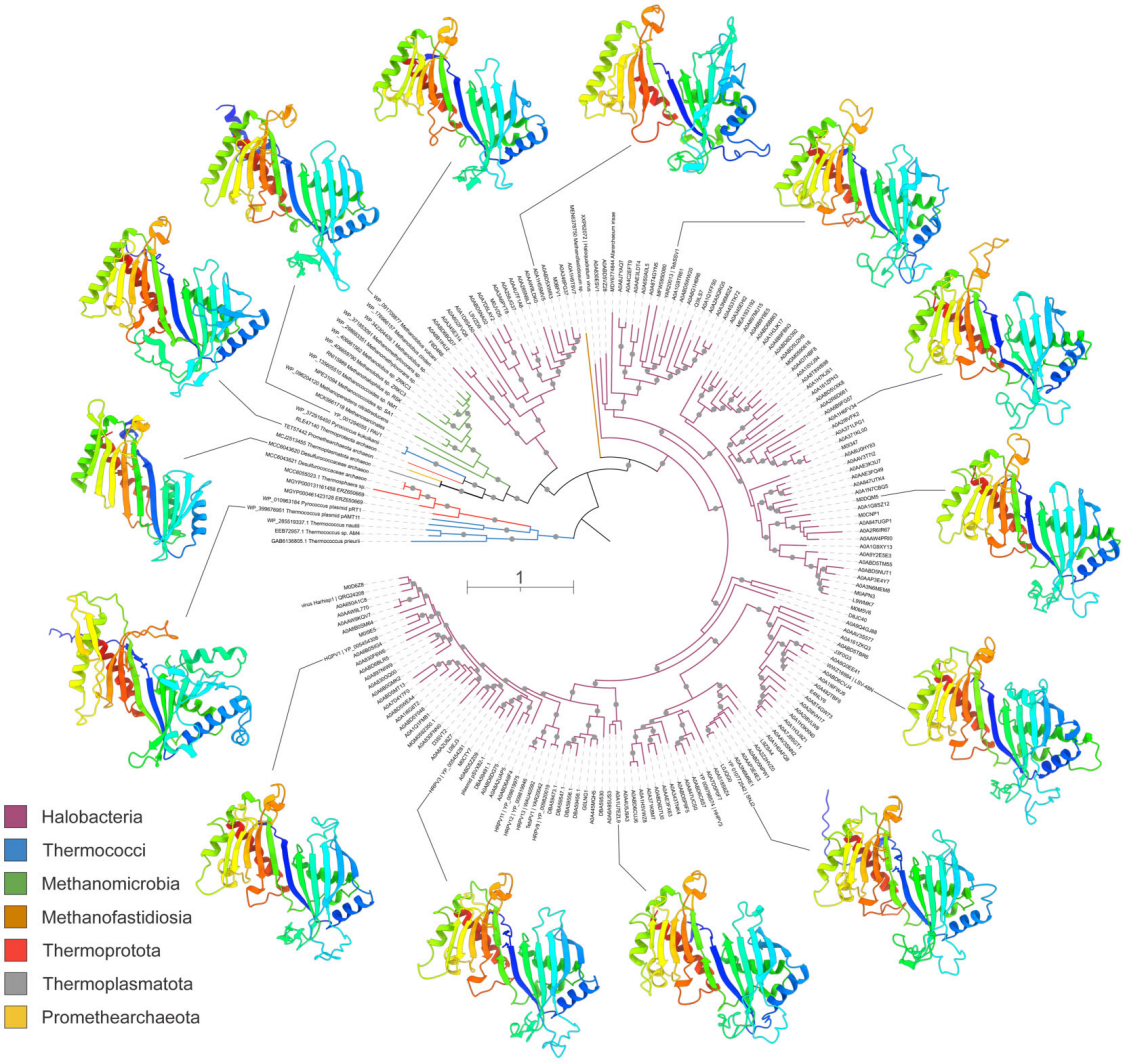
Maximum likelihood phylogeny and structural models of RepSNJ2 homologs from diverse archaea. Branches with SH-aLRT support values higher than 80% are indicated with grey circles. The branches are colored according to the taxonomic affiliation. Representative structural models of the N-terminal catalytic domains of RepSNJ2 homologs are shown using ribbon representation and colored using rainbow scheme from the N-terminus (blue) to C-terminus (red). The models are connected to the corresponding tips in the phylogeny. Taxa of the class Halobacteria are indicated with the corresponding protein accession numbers, whereas those from other archaeal lineages are indicated by accession numbers and species names. All viruses are indicated with the abbreviated names.

In the maximum likelihood phylogeny, the sequences formed several well-supported clades with nonhomogeneous taxonomic affiliation (Fig 6), suggesting occasional horizontal gene transfer between relatively distant archaeal lineages. Analysis of the structural models of DUF7845-domain proteins from different phylogenetic clusters confirmed that all of them contain a conserved TBP-like fold (Fig 6). Notably, all archaeal proteins contained the conserved N-terminal DUF7845 and the central wHTH domain, whereas the C-terminal domains were variable. For instance, homologs encoded by *Thermococcales* plasmids pAMT11 and pRT1 contained an additional wHTH domain. Overall, the broad taxonomic distribution and high sequence divergence of RepSNJ2 homologs compared to bacterial Rep_trans relatives suggest a long-term association of DUF7845-domain proteins with archaea.

Genomic context analysis using marker genes (e.g. capsid proteins, integrases, conjugation machinery components) showed that DUF7845-domain proteins are primarily associated with three MGE classes: (i) viruses and proviruses, (ii) plasmids and integrative elements, and (iii) integrative and conjugative elements (Supplementary Table S5). Notably, viruses encoding DUF7845-domain proteins belong to different families and can display either pleomorphic (e.g. SNJ2) or lemon-shaped (e.g. LSV-48N) virion morphologies, with the lemon-shaped viruses being associated not only with halophilic archaea [84], but also with hyperthermophilic, anaerobic archaea of the class *Thermococci* [85]. In phylogenetic analysis, DUF7845-domain proteins of thermococcal viruses (e.g. Pyrococcus abyssi virus 1 [85]) and plasmids (e.g. pAMT11 [86], pRT1 [87]) formed distinct clades. Notably, pAMT11 and pRT1 were shown to produce single-stranded replicative intermediates [86, 87], but in the absence of structural data the corresponding Reps were mistakenly considered to belong to the HUH superfamily, whereas the homolog encoded by PAV1 was annotated as a transcriptional regulator due to the presence of an identifiable wHTH domain [85].

**Figure 7.**
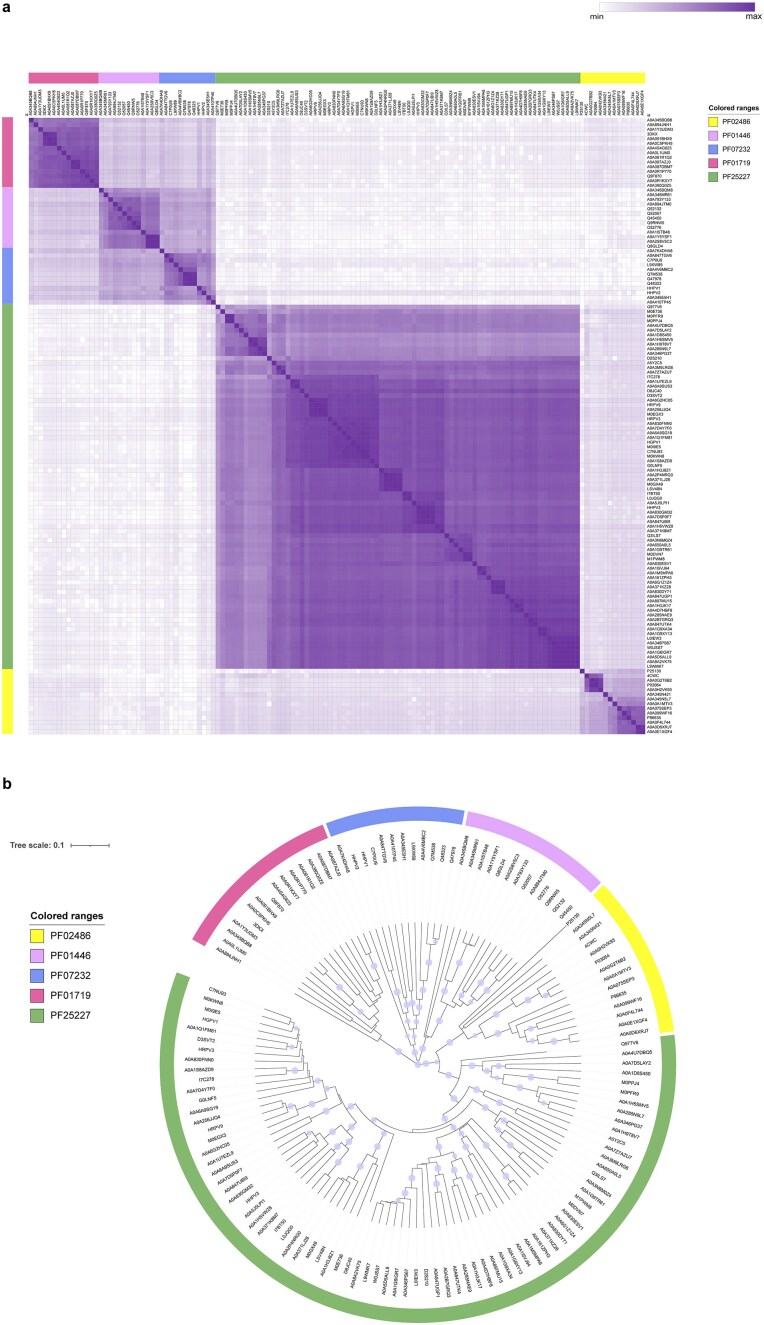
Global distribution and structural classification of the novel Rep-Arvir family. (**a**) Structural similarity matrix (heatmap) showing pairwise TM-scores between key domains of representative proteins from the PF25227 family and four known replication-related Pfam families (PF01446, PF07232, PF02486, PF01719). Proteins are grouped by family as indicated by the color-coded sidebar. Darker shades represent higher TM-scores, indicating greater structural similarity. (**b**) Structure-based dendrogram generated by hierarchical clustering of the proteins based on TM-scores of their key domains. Major branches corresponding to distinct Pfam families are colored consistently with panel (a).

Comparison and clustering of known or predicted protein structures provide an effective approach for classifying uncharacterized protein families [88]. To assess the evolutionary relationship between DUF7845-domain enzymes and RCR initiators from other families, the structural models of PF25227 were compared to those of PF02486 (exemplified by *S. aureus* plasmid pC221 RepD, PDB accession: 4CWC [7] and *Bacillus subtilis* ICE*Bs1* NicK, UniProt accession: Q96635) [89], PF01719 (*Streptococcus agalactiae* plasmid pLS1 RepB, PDB accession: 3DKX) [90], PF07232 (*Alphapleolipovirus* HHPV1 ORF1, UniProt accession: D3JVB8) [91], and PF01446 (*B. subtilis* plasmid pTA1060 Rep60, UniProt accession: Q45450) [92] (Supplementary Table S6). The resulting structural similarity matrix (Fig. 7a) revealed a clear intra-family clustering, forming five distinct similarity blocks that correspond closely with Pfam classifications. As expected, PF25227-domain proteins did not cluster with the structurally unrelated HUH superfamily Reps, and instead exhibited the strongest structural resemblance to the Rep_trans (PF02486) family. This relationship was further supported by hierarchical clustering based on structure-based similarity (Fig. 7b), in which PF25227 and PF02486 members formed a distinct super-cluster.

Together, these phylogenetic, genomic, and structural analyses establish the DUF7845-domain proteins as a distinct family of archaeal replication initiators, which we denote Rep-Arvir.

## Discussion

In many groups of archaeal viruses, including members of the *Betapleolipovirus* genus, the genome replication mechanisms remain unknown [30, 93]. Here, we dissected the replication strategy of the temperate archaeal virus SNJ2 and demonstrated that its integrase gene performs a dual function. First, it encodes an enzyme that catalyzes provirus integration and excision. Second, the integrase gene is an essential component of the replication operon that is formed only following genome excision. Excision of the provirus reorganizes the SNJ2 genome in such a way that a cluster of previously uncharacterized ORFs (*orf19*–*orf26*) is reconstituted into a single operon (Supplementary Table S1), where ORF19 (RepSNJ2) is proposed to be the key initiator of the RCR. The operon terminates by a conserved hairpin-like DNA structure, which corresponds to the putative origin of replication. Immediately adjacent to the *ori* is a short palindromic sequence (Fig. 3e), which we propose serves as the DNA-binding site of RepSNJ2, facilitating specific origin recognition and initiation of replication. This model resembles that proposed for the *S. aureus* plasmid RepDE protein [44, 50], a prototypical member of the Rep_trans family, which recognizes a plasmid-specific palindromic sequence and introduces a nick at a nearby conserved hairpin structure to initiate RCR. Despite the mechanistic parallels, there is no recognizable sequence similarity either between RepDE and RepSNJ2 or their recognition sequences. While the precise biochemical details of initiation remain to be fully elucidated *in vitro*, our cumulative genetic, structural, and biochemical data strongly support an RCR-like mechanism for SNJ2. The reconstitution of the replication operon upon excision safeguards the switch from lysogeny to active replication and represents an elegant regulatory mechanism of viral gene control that integrates genome topology with functional activation.

Every gene within the SNJ2 replication operon was found to be essential, implying that they act collectively as a coordinated replication module. HHpred analysis showed that genes in the reconstituted replication module encode DNA-binding proteins, putative endonuclease (ORF22) and a protein (ORF26) with homology to the Type III secretion system effector HopBA1, which possesses toxin activity. The presence of toxin and endonuclease genes in the replication module suggests that these components may function as toxin-antitoxin (addiction) modules, ensuring the stability of the replicon within the host population rather than being directly involved in the replication of the viral DNA. Although RepSNJ2 alone was sufficient for plasmid replication, the plasmid was unstable and gradually lost from the population in the absence of the accessory proteins.

RepSNJ2, the key component of the SNJ2 replication operon, is a novel RCR initiator. Using structure-based comparisons, we found that the N-terminal catalytic domain of ORF19 adopts the same TBP-like fold as the bacterial members of the Rep_trans family (PF02486), which were previously considered to be restricted to bacterial MGEs, including plasmids (e.g. *Staphylococcal* pT181 family [7]), filamentous bacteriophages (e.g. CTXΦ, which carries cholera toxin genes), and conjugative transposons [7, 89, 94–96]. Rep_trans proteins are structurally and evolutionarily unrelated to the other families of RCR initiators of the HUH superfamily, such as Rep_1 (PF01446) and Rep_2 (PF01719) [11, 75]. This finding provides the first evidence of an archaeal viral protein adopting the TBP-like fold for replication, thereby expanding the evolutionary reach of this superfamily beyond the bacterial domain. Notably, in structure-based comparisons Rep_trans and Rep-Arvir form separate, well delineated clusters, arguing against the recent interdomain horizontal gene transfer. Instead, it appears likely that the common ancestor of these enzymes has already existed at the time of the last universal cellular ancestor and has independently diversified and spread within MGEs of bacteria and archaea. Results of the structure-based clustering provided strong support for recognition of Rep-Arvir as a novel, cohesive family of archaeal replication initiators, distinct from but evolutionarily related to the bacterial Rep_trans family.

Mutagenesis analyses confirmed that Y283, the predicted catalytic tyrosine, is essential for replication initiation, consistent with the nucleophilic mechanism of canonical Rep_trans proteins. The acidic residues E25 and E303 were also indispensable, whereas D207 played only a minor structural role, possibly stabilizing the catalytic pocket under high-salt conditions characteristic of haloviruses. Notably, multiple sequence alignment of RepSNJ2 homologs from betapleolipoviruses, showed that in HGPV1 and HRPV3 E303 is replaced by a glycine. This nonconservative substitution cannot perform the same charge-based function as E303. It is possible that HGPV1 and HRPV3 have evolved a compensatory acidic residue at a different structural position. These observations suggest that while the core catalytic mechanism is conserved, subtle adaptations may optimize enzymatic function under extreme ionic strengths.

A defining, conserved feature of the Rep-Arvir family, not observed in the bacterial Rep_trans counterparts, is the fusion of the catalytic endonuclease domain with the MarR-like wHTH domain. The latter domain could mediate specific recognition of the conserved hairpin, which functions as the viral origin. Disruption of this domain (G410A) completely abolished replication, highlighting its critical role in origin recognition and binding (Fig. 4d). Collectively, these findings support a mechanistic model in which MarR-like domain recognizes the hairpin origin and positions the catalytic core for site-specific nicking. The resulting covalent attachment of RepSNJ2 to the 5′ end of the nicked DNA releases the 3′-OH group which likely primes the RCR.

As previously reported, betapleolipovirus genomes packaged within viral particles frequently harbor inherent ssDNA nicks and short single-stranded regions [41, 68]. However, the biological significance of these structural features remains elusive, particularly regarding their presence or function during active viral replication. Importantly, these previously described short discontinuities are unlikely to account for the ssDNA species detected in our Southern blot analysis. Under the neutral conditions used in our assay, such short single-stranded patches would remain tightly annealed to the complementary strand and fail to dissociate into transferable, free ssDNA, preventing probe hybridization (Fig. 5c). Furthermore, the specific ssDNA signal we detected resolved as a clearly defined, faster-migrating low-molecular-weight band, distinct from the high-molecular-weight band representing the full-length viral genome (Fig. 2b, lane 3). This clear contrast in both electrophoretic mobility and probe accessibility provides physical evidence that the specific signal detected under neutral conditions corresponds to *bona fide* ssDNA replication intermediates, rather than bulky, nicked double-stranded viral genomes.

While these findings strongly support an RCR mechanism, the direct involvement of ORF19 in the enzymatic cleavage of the *ori* and ligation of the extruded ssDNA intermediate remain to be definitively established. Direct analysis of ssDNA intermediates in *orf19* mutants *in vivo* is not possible because ORF19 is strictly essential for replication. Indeed, loss-of-function mutations completely abolish plasmid replication, making it technically unfeasible to obtain sufficient replicating DNA to capture this arrested state *in vivo*. Therefore, while our data perfectly align with the RCR model, further *in vitro* biochemical characterization will be necessary to definitively confirm whether ORF19 directly possesses the postulated endonuclease activity.

The Rep-Arvir family has a broad distribution in archaea that thrive in diverse ecological settings. It is particularly abundant within the phylum *Methanobacteriota*, predominantly in the class *Halobacteria*, including the basal lineage, *Candidatus* Afararchaeaceae [83], but also present in methanogenic and hyperthermophilic archaea of the classes *Methanomicrobia* and *Thermococci*, respectively. However, although less common, Rep-Arvir representatives were also detected in archaea of the phyla *Thermoproteota, Thermoplasmatota* and *Promethearchaeota* (Asgard archaea). We note that a single homolog (A0A523VRS9) was identified in Asgard archaea thus far (Supplementary Table S4 and  Supplementary Fig. S9) and further exploration of the Asgard archaeal mobilome is needed to substantiate this observation.

The Rep-Arvir family has been adopted by diverse archaeal MGEs, including morphologically distinct pleomorphic and lemon-shaped viruses [84], plasmids as well as integrative and conjugative elements. The presence of the same replication module across such a diverse array of MGEs strongly suggests that it functions as a highly successful replication cassette. The pervasive association of Rep-Arvir with distinct MGE classes epitomizes the modular evolution of archaeal mobilome [97].

Within *Halobacteria*, the Rep-Arvir proteins encoded by all currently ICTV-recognized betapleolipoviruses are highly conserved, and their downstream noncoding regions consistently harbor highly conserved stem–loop structures preceded by more variable short palindromic motifs. Similar downstream structural organizations are also observed in some plasmids encoding related Rep-Arvir proteins (Supplementary Fig. S10). This bipartite arrangement resembles the organization of RCR origins in bacterial systems, in which a conserved structural element is associated with a more variable Rep-recognition region [98, 7]. By contrast, outside *Halobacteria*, we found no convincing evidence for an equally conserved downstream sequence/structure element. Although palindromic or hairpin-forming sequences can be detected near some nonhaloarchaeal *rep-arvir* genes, these features are not consistently conserved with respect to sequence, spacing, or position relative to the gene. Moreover, previous studies on thermococcal rolling-circle plasmids pRT1 and pAMT11 have suggested that the relevant origin-associated region may instead be located upstream of the rep gene [86, 87]. Together, these observations suggest that conserved downstream structural elements are a common feature of *rep-arvir* loci in viruses and plasmids within *Halobacteria*, whereas their broader evolutionary conservation and functional relevance in other archaeal lineages remain to be established.

In summary, through a combination of structural modeling, structure-based comparisons, and experimental validation, we have discovered a previously unrecognized family of replication proteins widespread in archaeal MGEs. However, for many archaeal viruses, including certain members of *Pleolipoviridae* [39], the replication mechanisms and the associated proteins remain unknown. We anticipate that the integrative computational and experimental approaches described herein will facilitate the discovery of the full diversity of replication strategies employed by the archaeal mobilome.

## Supplementary Material

gkag363_Supplemental_Files

## Data Availability

All data generated or analyzed during this study are included in this published article and its supplementary information files.
