# Supplementary material for: A new family of rolling-circle replication endonucleases widespread in archaeal viruses and plasmids
Source: Nucleic Acids Res. 2026 Apr 24;54(8):gkag363. doi: 10.1093/nar/gkag363 (PMC13107129; doi:10.1093/nar/gkag363)
Supplement: gkag363_Supplemental_Files [file gkag363_supplemental_files.zip › Revised_Supplementary_materials_Clean.doc]

**SUPPLEMENTARY INFORMATION FOR:**

**A new family of rolling circle replication endonucleases widespread in archaeal viruses and plasmids**

**Authors**

Yixuan Wang^1^, Zihao Duan^1^, Zhao Chen^1^, Shuyu Li^1^, Huiyi Chen^1^, Jiangling Chen^1^, Yangyang Wang^1^, Jiaying Wang^1^, Kang An^1^, Jialin Xiang^1^, Shishen Du^1^, Ying Liu^1^, Mart Krupovic^2*^ and Xiangdong Chen^1*^

**Affiliation**

1 State Key laboratory of Virology, College of Life Sciences, Wuhan University,

China, 430072

2 Institut Pasteur, Université Paris Cité, CNRS UMR6047, Cell Biology and Virology of Archaea Unit, 75015 Paris, France

*** To whom correspondence should be addressed:**

Mart Krupovic, e-mail: [mart.krupovic@pasteur.fr](mailto:mart.krupovic@pasteur.fr)

Xiangdong Chen, e-mail: xdchen@whu.edu.cn

**Supplementary Materials and Methods**

**Strain construction**

The deletion or mutation of *orf*xSNJ2 in *Natrinema* sp. J7-1-F was performed as previously described (1). Briefly, the knockout vectors pNBK-ORFx were transformed into J7-1-F. Allelic single exchange screening was conducted using Hv-Ca medium. The transformants were subsequently subcultured in MGM medium to facilitate secondary homologous recombination. Cultures were then plated on MGM plates containing 5-fluoroorotic acid (5-FOA) at a final concentration of 0.25 mg/mL to select for deletion recombinants (2). At least three independent clones of each strain were confirmed by PCR using specific primer pairs. The correctness of the introduced mutations was further verified by subjecting these PCR products to Sanger sequencing.

**Plasmid construction.**

Six classes of plasmids were constructed and categorized based on their functionality. Primers used in this study are listed in Supplementary Table 7.

(A) The plasmid pNBK-F, which contains the upstream and downstream flanking sequences of the target genes, was used to construct the mutant strains. These include the J7-1-F ORFxSNJ2 point mutants (where x = 1, 19-26) and the J7-1-F SNJ2 Int^Mut^ strain, in which premature stop codons were introduced at positions 88 and 90 of the integrase gene. Overlap extension PCR was performed using primer pairs *orf*xM-F1/R1 and *orf*xM-F2/R2 with the SNJ2 viral genome as a template to amplify the upstream and downstream homologous arms of the mutations. The resulting PCR products were ligated into *Hind*III-*Afl*II digested pNBK-F using the 2× MultiF Seamless Assembly Mix (ABclonal, Wuhan, China).

(B) Complementary plasmids. To construct pYCJ2260, PCR amplification was performed using the primer pairs 2260-F and 2260-R with circularized SNJ2 genome as a template to amplify the complementary segments which carries the complete integrase gene operon (*orf1*-*3* of SNJ2). The resulting PCR products were ligated into BamH*I*-Hind*III* digested pYCJ using the 2× MultiF Seamless Assembly Mix (ABclonal, Wuhan, China).

(C) The pSNJ2Int^Mut^ plasmid was constructed using the circularized SNJ2 viral genome as the template. A fragment spanning nucleotides 6556-2066 of the SNJ2 circularized genome (12,503 bp) was amplified by segmented PCR. Two early stop codons were introduced into the integrase gene at *both* amino acid positions Arg88 and Gln90 by high-fidelity PCR using a pair of mutagenic primers (pSNJ2-M-F, pSNJ2-M-R). The amplified mutant fragment was assembled with a 3,645-bp backbone fragment excised from the pFJ6-MCS plasmid using *SnaB*I and *Not*I. This backbone contained the *Escherichia coli* origin of replication (*ori*), the ampicillin resistance gene (*AmpR*), and the uracil biosynthesis gene (*pyrF*). The assembly was performed using the 2× MultiF Seamless Assembly Mix (ABclonal, Wuhan, China) according to the manufacturer’s instructions. The resulting recombinant plasmid was verified by Sanger sequencing.

Other truncated derivatives of pSNJ2Int^Mut^ were constructed by PCR amplification of shortened genomic fragments corresponding to the desired regions, followed by assembly into the same backbone vector using the same procedure.

The Mev-resistant plasmid was constructed by assembling a Mev-resistant (*mevR*) fragment, amplified from pYC-SHS, with an 8,843-bp backbone fragment derived from pSNJ2Int^Mut^P3-19(+) by *Nde*I and *Mfe*I digestion, using the same assembly method as described above.

(D) To introduce specific mutations into the *orf19* gene within the pSNJ2Int^Mut^P3-19(+) plasmid, a seamless cloning-based mutagenesis strategy was employed. The pSNJ2Int^Mut^P3-19(+) plasmid was first digested with *Sph*I, yielding two distinct fragments (6341 bp and 3701 bp). The 6341 bp fragment was gel-purified and served as the vector backbone. For each targeted mutation (taking E25A as a representative example), two overlapping PCR fragments containing the point mutation were generated. The upstream fragment was amplified using the universal forward primer *Sph*I-24-F and the mutation-specific reverse primer ORF19-E25A-R, whereas the downstream fragment was amplified using the mutation-specific forward primer ORF19-E25A-F and the universal reverse primer *Sph*I-ORF19-R. Finally, these two mutated overlapping fragments were co-ligated into the *Sph*I-digested 6341 bp backbone using the 2× MultiF Seamless Assembly Mix (ABclonal, Wuhan, China). All other *orf19* mutant plasmids were constructed following this identical procedure using their corresponding mutagenic primer pairs listed in Supplementary Table S9.

(E) To construct the plasmids expressing *orf19* and its truncated variants under the control of the P_phaR_P promoter, two separate DNA fragments were amplified. First, the P_phaR_P promoter was amplified using the pRF plasmid as a template (3). Concurrently, the *orf19* gene and its corresponding downstream sequences were amplified from the SNJ2 genome using specific primers. Each truncated 19-xbp segment was amplified using the primer pairs ORF19-xbp-F/ ORF19-xbp-R (x =90,180,192,198,204,210,231) from SNJ2 genome. These two fragments were ligated into the *NotI* and *StuI* digested linearized plasmid using the 2× MultiF Seamless Assembly Mix (ABclonal, Wuhan, China).

(F) α-Amylase reporter plasmids: To construct pSNJ2Int^Mut^P3-19(+)-Hpro-*amyH*, overlap extension PCR was performed using the primer pair Hpro-*amyH*-F1/R1 with pFJ6-Hpro-*amyH* as templates to amplify the H promoter and the *amyH* gene (4, 5). The resulting PCR products were ligated into the *Not*I and *Hind*III-digested pSNJ2Int^Mut^P3-19(+) plasmid using the 2× MultiF Seamless Assembly Mix (ABclonal, Wuhan, China).

(G) To construct the expression plasmids pFJ6-P-ORF26-GFP and pFJ6-P-ORF19-His (along with its mutant variants), a seamless assembly strategy was employed using the 2× MultiF Seamless Assembly Mix (ABclonal, Wuhan, China). The P_phaR_P promoter and GFP gene sequence were amplified using the pRF plasmid as a template(3). For the pFJ6-P-ORF26-GFP construct, three fragments were generated and co-ligated into the *Mun*I-*Sph*I-digested pFJ6 backbone: the P*_phaR_P* promoter (amplified with primers *Mun*I-P-F/P-ORF26-R), the *orf26* gene amplified directly from the SNJ2 viral genome (P-ORF26-F/ORF26-GFP-R), and the GFP sequence amplified from the pRF plasmid (ORF26-GFP-F/*Sph*I-ORF26-R). For the pFJ6-P-ORF19-His construct and its derivatives, two fragments were assembled into the *Mun*I-*Afl*II-digested pFJ6 backbone: the P*_phaR_P* promoter (amplified with primers *Mun*I-P-F/P-19-R), and the wild-type and mutant *orf19*-His sequences were amplified from the pSNJ2Int^Mut^P3-19(+) plasmid and the corresponding *orf19* mutant plasmids, respectively, using the specific primer pair P-19-F/*Afl*II-ORF19His-R. All primers utilized in these constructions are detailed in Supplementary Table S9.

**Supplementary Text**

**The SNJ2-based shuttle vector expands the available genetic tools for *Natrinema* sp. J7 strains**

The haloarchaeon *Natrinema* sp. J7-1 harbors two temperate viruses: SNJ2, a member of the *Pleolipoviridae* family that is integrated into the host chromosome, and SNJ1, a member of the *Simuloviridae* family that persists as a cytoplasmic circular double-stranded DNA element (pHH205) (6, 7). In our previous work, we established a series of *Natrinema* sp. J7-derived strains carrying different combinations of these viruses or plasmids, and developed two *Natrinema*-*E. coli* shuttle vectors: the SNJ1-based pYC series and pFJ6, which is based on a chromosomal autonomous replication region of *Natrinema* sp. J7 (4, 8). These tools have supported in-depth studies on interactions between the two coexisting viruses and between viruses and the host (5-8).

As described earlier, the SNJ2 provirus is present in both *Natrinema* sp. J7-1 (which carries SNJ1) and in strain CJ7 (which lacks any plasmids). However, upon mitomycin C (MMC) induction, the level of SNJ2 excision and release from CJ7 was markedly lower than from J7-1, but, when the SNJ1-based plasmid pYC-SHS was introduced into CJ7, the SNJ2 release level after MMC treatment increased to match that of J7-1 (7). This observation suggests a virus-virus interaction dependent on the SHS region of SNJ1. The SNJ2-derived plasmid pSNJ2Int^Mut^P3-19(+), which contains the minimal replication region of SNJ2, provides a useful system for genetically dissecting such interactions.

Importantly, to definitively confirm that the engineered shuttle plasmid replicates autonomously as episomes rather than integrating into the host chromosome, we performed two independent verification assays. First, to rule out site-specific integration for the integrase-mutated constructs pSNJ2Int^Mut^ and pSNJ2Int^Mut^P3-19(+), PCR analysis targeting the *attL* junction was performed. This analysis yielded no specific amplification products, confirming the absence of site-specific integration (Supplementary Figure S4a). (Note: The pSNJ2-P-ORF19(+), lacks the integrase gene entirely, precluding the need for this specific assay). Second, to verify the extrachromosomal state of pSNJ2Int^Mut^, pSNJ2Int^Mut^P3-19(+) and pSNJ2-P-ORF19(+) and rule out homologous recombination, a plasmid rescue assay was performed. As expected, the plasmids successfully rescued into *E. coli* exhibited restriction digestion patterns entirely identical to those of the original input vectors (Supplementary Figure S4b). Furthermore, these rescued plasmids retained their biological functionality and were successfully back-transformed into haloarchaeal cells. Together, these results confirm that the plasmids are stably maintained as free- replicating episomes without chromosomal integration or structural rearrangements.

To expand the utility of this system, we constructed a derivative of pSNJ2Int^Mut^P3-19(+) by replacing the original *pyrF* selection marker (uracil auxotrophy) with *hmgA*, which confers resistance to mevinolin (Mev), yielding a vector conferring Mev resistance *mevR*-pSNJ2Int^Mut^P3-19(+) (Figure S3b). We first assessed its compatibility with existing shuttle vectors. As shown in Figure S4A, co-transformation of J7-3-F with pYC-SHS and pSNJ2Int^Mut^P3-19(+), or pFJ6-MCS and *mevR*-pSNJ2Int^Mut^P3-19(+), led to stable maintenance and segregation of both plasmids during host cell division. Quantitative PCR (qPCR) further confirmed consistent plasmid copy numbers after multiple rounds of subculturing in antibiotic-free medium (Figure S4c/d). These results indicate that SNJ2-derived vectors bearing distinct selection markers are fully compatible with previously established shuttle systems and are stably inherited.

To assess the host range, we introduced *mevR*-pSNJ2Int^Mut^P3-19(+) into three strains of *Natrinema* sp.: *N. gari* JCM 14663, *N. versiforme* JCM 10478, and *N. pellirubrum* JCM 10476. Among them, *N. gari* JCM 14663 exhibited the highest transformation efficiency (3.1×10^3^ cfu/μg DNA). The plasmid was stably maintained in *N. gari* during both selective and non-selective cultivation, with plasmid copy numbers being even higher than those in *Natrinema* sp. J7 (data not shown). In contrast, *N. versiforme* and *N. pellirubrum* harbored endogenous *hmgA* genes, rendering Mev selection ineffective, as evidenced by colony formation on selective plates by negative control strains. The vector also failed to replicate in non-*Natrinema* haloarchaea, such as *Haloarcula hispanica* 33960 and *Haloferax volcanii* DS52, indicating a limited host range.

To test its capacity for heterologous gene expression, we inserted a multiple cloning site (MCS) into pSNJ2Int^Mut^P3-19(+) and cloned the amylase gene from *Haloarcula hispanica* DSM 4426 under control of the strong *hsp70* promoter. Transformation of the resulting construct into J7-3-F yielded 12 randomly selected transformants, all of which produced clear starch hydrolysis zones on starch-containing minimal medium (Figure S4d). By contrast, transformants carrying the empty vector exhibited no amylase activity, while the non-transformed control strain failed to grow on the selective starch medium.

In addition, a previous study showed that expressing a 1-656 bp fragment of SNJ1 ORF4 from the pFJ6 vector conferred superinfection immunity in CJ7 by preventing SNJ1 reinfection (4). To validate whether the SNJ2-derived system could also express functional viral genome elements, we expressed the same ORF4 fragment using pSNJ2Int^Mut^P3-19(+). As shown in Figure S4e, expression of this fragment conferred robust superinfection immunity in CJ7, mirroring the effect observed with the pFJ6-based system.

Taken together, these results demonstrate that the SNJ2-based shuttle vector pSNJ2Int^Mut^P3-19(+) is genetically stable, compatible with existing vectors, effective for heterologous gene expression, and capable of expressing functional viral elements. It thus represents a powerful and versatile addition to the genetic toolkit available for studying *Natrinema* sp. J7 strains and their proviral elements.

**Supplementary Dataset S1. Multiple sequence alignment of DUF7845-containing homologs.** This dataset provides the full alignment of protein sequences used for the phylogenetic analysis in this study. (Provided as an Excel file)

**SI APPENDIX, TABLE S1-S9**

**Supplementary Table 1: Gene arrangement and intergenic distances within the extended operon of circularized SNJ2**

| ORF ID | Length(bp) | Relative position | Overlap/Gap with next ORF |
| --- | --- | --- | --- |
| *orf19* | 1980 | Right terminus | 40-bp overlap with *orf20* |
| *orf20* | 201 | - | 2-bp gap with *orf21* |
| *orf21* | 537 | - | 4-bp overlap with *orf22* |
| *orf22* | 522 | - | 4-bp overlap with *orf23* |
| *orf23* | 120 | - | 4-bp overlap with *orf24* |
| *orf24* | 333 | - | 8-bp overlap with *orf25* |
| *orf25* | 240 | - | 13-bp overlap with *orf26* |
| *orf26* | 240 | Formed only after genome circularization and is located downstream of the integrase (ORF1). | 2-bp gap with *orf1* (int^SNJ2^) |
| *orf1* | 1050 | Left terminus which contains the promoter region of the replication operon (*orf19-26*). | co-transcribed with *orf2-3* |
| *orf2* | 336 | - | 8-bp overlap with *orf3* |
| *orf3* | 326 | Located upstream of *orf1*, involved in the regulation of integrase expression. | - |

**Supplementary Table 2: Protein function prediction using HHpred**

| protein | Hit | Putative function | Probability | E-value |
| --- | --- | --- | --- | --- |
| ORF3  141aa | 3ZMD_C | Putative transcriptional regulator; transcription, MarR-family, winged helix motif; {*Streptomyces coelicolor*} | 99.4 | 7.4e-12 |
| ORF2  111aa | / | Unknown function | / | / |
| ORF1  349aa | 1A0P_A | Site-specific recombinase XerD; {*Escherichia coli*} SCOP: d.163.1.1, a.60.9.1 | 100 | 1.1e-32 |
| ORF26  79aa | [/](http://www.rcsb.org/pdb/explore/explore.do?structureId=5T09) | Unknown function | / | / |
| ORF25  79aa | 8Q5B_A | Small CPxCG-related zinc finger protein; zinc-finger, {*Haloferax volcanii DS2*} | 96.76 | 0.0032 |
| ORF24  110aa | / | Unknown function | / | / |
| ORF23  39aa | 8GQD_E | Protein-arginine kinase activator protein; Protein quality control, Zinc finger, Enzyme activation, {*Staphylococcus aureus*} | 92.54 | 0.34 |
| ORF22  173aa | 8RPX_E | Restriction endonuclease (*Nho*I); DNA endonuclease, DNA modification | 97.05 | 0.032 |
| ORF21  178aa | / | Unknown function | / | / |
| ORF20  66aa | 4AYB_P | DNA-directed RNA polymerase subunit Rpo12; Zn-binding protein; {*Sulfolobus shibatae*} | 83.2 | 0.89 |
| ORF19  659aa | 7BZH_A | Sul7s; Crenarchaeal DNA binding protein {*Sulfolobus islandicus*} | 96.67 | 0.0083 |

"/" indicates that no significant hits were found.

**Supplementary Table 3: Distribution of the conserved downstream DNA motif in halophilic archaeal genomes.**

The table lists representative BLASTn hits for the second conserved DNA motif. Each entry includes the motif coordinates, sequence identity, and proximity to ORF19-like genes. Genomic contexts, such as association with proviral regions or integrated elements (IEs), are also indicated. The whole sequence alignments for *Haloarcula marismortui* ATCC 43049 and *Haloferax volcanii* DS2 (Halfvol1 provirus) with Betapleolipovirus are shown in Figure 3d.

**Supplementary Table 4: Representative Rep-Arvir family homologs identified in diverse archaeal phyla.**

| Protein Description | Accession | Source | E value | Per. Ident | Acc. Len | Phylum/Kingdom | doamin |
| --- | --- | --- | --- | --- | --- | --- | --- |
| MAG: hypothetical protein E3J52_10455  [*Promethearchaeota archaeon*] | TET57442.1 | marine sediment metagenome | Query | 100.00% | 518 | *Promethearchaeota* | Archaea |
| hypothetical protein  [*Candidatus Afararchaeum irisae*] | MDY6774844.1 | salt lake metagenome | 5e-26 | 15.09% | 658 | *Nanobdellati*  (Kingdom) | Archaea |

Homologs were identified using an iterative PSI-BLAST search against the NCBI non-redundant protein database, with the sequence of TET57442.1 from a *Promethearchaeota archaeon* used as the initial query. The table lists representative hits from distinct phyla, showing the NCBI accession number, E-value, and percent sequence identity relative to the query.

**Supplementary Table 5: List of 527 protein sequences annotated with the PF25227 domain retrieved from the InterPro database.** (Provided as an Excel file)

**Supplementary Table 6: Details of conserved domain regions extracted from AlphaFold-predicted structures of representative proteins based on Pfam-defined boundaries.** (Provided as an Excel file)

**Supplementary Table 7: Strains used in this study**

| Strains | Description | Source and reference |
| --- | --- | --- |
| *Natrinema* sp. J7-1 | lysogenic strain of SNJ1 and SNJ2 | (4, 6, 7) |
| *Natrinema* sp. J7-1-F | J7-1Δ*pyrF*, and lysogenic strain of SNJ1 and SNJ2 | (4) |
| *Natrinema* sp. J7-1-FInt^Mut^ | Integrase mutation strain of J7-1-F | This study |
| *Natrinema* sp. J7-1-FInt^Mut^ /pYCJ2260 | J7-1-FInt^Mut^ containing complemented vector pYCJ2260 | This study |
| *Natrinema* sp. J7-1-FΔint*::pyrF* | J7-1Δ*pyrF*Δint^SNJ2^*::pyrF* | (2) |
| *Natrinema* sp. J7-1-FΔint*::pyrF*/pYCJ2260 | J7-1-FΔint*::pyrF* containing complemented vector pYCJ2260 | This study |
| *Natrinema* sp. J7-1-FΔ*orf*x | J7-1Δ*pyrF*Δ*orf*x^SNJ2^(x=19,20,21,22,23,24,25,26) | This study |
| *Natrinema* sp. J7-3-F | Detection of plasmid stability | (2) |
| *Natrinema* sp. CJ7-F | Detecting plasmid expression activity | (8) |
| *Haloferax vocanii* H26 | Host range of shuttle vectors | (12) |
| *Natrinema pellirubrum JCM 10476* | Host range of shuttle vectors | CGMCC |
| *Natrinema Versifome* JCM 10478 | Host range of shuttle vectors | CGMCC  1.2365 |
| *Natrinema gari JCM 14663* | Host range of shuttle vectors | Gift from L.Cui |
| *Escherichia coli* DH5α | Cloning host for all newly constructed plasmids. s*upE*44 Δ*lacU*169(ϕ80*lacZ*ΔM15) *hsdR*17 *recA*1 *endA*1 *gyrA*96 *thi*-1 *relA*1 | CCTCC^b^ |
| *Escherichia coli* JM110 | For plasmid demethylation. *dam dcm supE*44 *hsdR*17 *thi leu rpsL lacY galK galT ara tonA thr tsx* Δ(*lac*-*proAB*) F’ (*traD*36*proAB*+ *lacIq lacZ* ΔM15) | CCTCC |

a CMGCC, China General Microbiological Culture Collection Center

b: CCTCC, China Center for Type Culture Collection.

**Supplementary Table 8**: Plasmids used in this study

| Plasmids | Description | Source and reference |
| --- | --- | --- |
| pFJ6-MCS | An *E. coli-Natrinema* shuttle vector based on replication element of CJ7 chromosome, containing pUC19, *pyrF* marker and multiple cloning site. | (8) |
| pNBK-F | Haloarchaeal suicide vector containing the halobacterial mevinolin (Mev) resistance gene and *pyrF* gene with P_fdx_ promoter | Gift from Y. Huang |
| pYC-SHS | An *E. coli-Natrinema* shuttle vector based on replication element of SNJ1 genome, containing pUC19, *mevR* marker and multiple cloning site. | (4) |
| pYCJ-2260 | pYCJ digested with *BamH*I-*Hind*III fragment containing P_3_ promoter of Int^SNJ2^ *orf1*-*3* genes | This study |
| pNBK-Int^Mut^ | pNBK-F with *Hind*III-*Afl*II fragment containing flanking homologous of Int^Mut^ | This study |
| pNBK-ORFx | pNBK-F with *Hind*III-*Afl*II fragment containing flanking homologous of ORFx（x=19,20,21,22,23,24,25,26） | This study |
| pFJ6-P-ORF19-His | pFJ6-MCS derivative expressing C-terminal His-tagged ORF19 under the control of the *Haloferax mediterranei* P*_phaRP_* promoter (inserted between the *Mun*I and *Afl*II sites). | This study |
| pFJ6-P-ORF19(X)-His | Same as pFJ6-P-ORF19-His, but harboring specific mutations within *orf19* (X = E25A, D207A, Y283A, Y283F, E303A, G410A, or D408A/G409A/G410A). | This study |
| pFJ6-P-ORF26-GFP | pFJ6-MCS derivative expressing C-terminal GFP-tagged ORF26 under the control of the *Haloferax mediterranei* P*_phaRP_* promoter (inserted between the *Mun*I and *Sph*I sites). | This study |
| pSNJ2Int^Mut^ | An *E. coli-Natrinema* shuttle vector based on fragment of SNJ2 genome (*orf11*-*26*, *orf3*-*1*), containing pUC19, *pyrF* marker and multiple cloning site. | This study |
| pSNJ2Int^Mut^Δ*orfx* | Gradually knockout the *orf11*-*21* of pSNJ2Int^Mut^ | This study |
| pSNJ2Int^Mut^P3-19(+) | pSNJ2Int^Mut^ with *orf19*-*26 and orf3*-*1* fragment | This study |
| pSNJ2Int^Mut^P3-19(-) | pSNJ2Int^Mut^ with *orf19*-*26 and orf3*-*1* fragment, without the downstream sequence of *orf19* | This study |
| pSNJ2Int^Mut^P3-19(x bp) | pSNJ2Int^Mut^P3-19(+) with the truncation of the downstream sequence of *orf19* | This study |
| pSNJ2-P-19(x bp) | Containing promoter of the phaRP operon from *Haloferax mediterranei* and *orf19,* with the truncation of the downstream sequence of *orf19* | This study |
| pSNJ2-P-19M-270 bp | pSNJ2-P-19-270 bp with a nonsense mutation of *orf19* | This study |
| pSNJ2-19-270 bp | pSNJ2-P-19-270 bp with the promoter was removed | This study |
| pSNJ2Int^Mut^P3-19(+)-xA | pSNJ2Int^Mut^P3-19(+) with Alanine mutations in conservative amino acids. (include E25A, D207A, K281A, H282A, Y283A, Y283F, Y284A, M285A, E303A, D408A, D409A, G410A, 408-410A) | This study |
| pSNJ2Int^Mut^P3-19(+)-*amyH* | pSNJ2Int^Mut^P3-19(+) containing promoter and *amyH* gene | This study |
| 1-656-pSNJ2Int^Mut^P3-19(+) | pSNJ2Int^Mut^P3-19(+) containing the superinfection immunity fragment of SNJ1 | This study |
| *mevR*-pSNJ2Int^Mut^P3-19(+) | pSNJ2Int^Mut^P3-19(+) replace the *pyrF* gene with the *mevR* resistance gene | This study |

**Supplementary Table 9**: Primers used in this study

| Primers | sequences (5'-3') | Description |
| --- | --- | --- |
| pNBK-19M-F1 | GCGGCGGCCGCTCCGCGAGTAAGCTTaccgaaggccgccgcggc | primers used for the *orf19M* strain, J7-1-F *orf19M* construction |
| pNBK-19M-R1 | gtgagcgcgcagTagttcctccggttt |  |
| pNBK-19M-F2 | aaaccggaggaactActgcgcgctcac |  |
| pNBK-19M-R2 | AGTCCGTAATACGACTCACTTAAGtcggtcgacttcgagcgccg |  |
| pNBK-20M-F1 | GGCGGCCGCTCCGCGAGTAAGCTTccagcgcggccggaacgt | primers used for the *orf20*M strain, J7-1-F *orf20*M construction |
| pNBK-20M-R1 | gccttcggcctcTagccgcaggactcg |  |
| pNBK-20M -F2 | cgagtcctgcggctAgaggccgaaggc |  |
| pNBK-20M -R2 | AGTCCGTAATACGACTCACTTAAGgagcccgccgatggggat |  |
| pNBK-21*M* -F1 | agtagccgaaacggggtgagcgcgcagaag | primers used for the *orf21*M strain, J7-1-F *orf21M* construction |
| pNBK-21*M* -R1 | ggcgtgaccgtcTgaacgacctacttc |  |
| pNBK-21*M* -F2 | gaagtaggtcgttcAgacggtcacgcc |  |
| pNBK-21*M* -R2 | AGTCCGTAATACGACTCACTTAAGagcccgaaggccagcggtga |  |
| pNBK-22M-F1 | GCGGCGGCCGCTCCGCGAGTAAGCTTcgttcggacggtcacgcc | primers used for the *orf22*M strain, J7-1-F *orf22M* construction |
| pNBK-22M-R1 | gttatgagttctTgagcgtcggaactc |  |
| pNBK-22M-F2 | gagttccgacgctcAagaactcataac |  |
| pNBK-22M-R2 | AGTCCGTAATACGACTCACTTAAGgcagcgggggtcgaggtgtc |  |
| pNBK-23M-F1 | GCGGCGGCCGCTCCGCGAGTAAGCTTgtaccaccgcccggcgag | primers used for the *orf23*M strain, J7-1-F *orf23M* construction |
| pNBK-23M-R1 | tgaccgatgagcTgactcgtcgagaac |  |
| pNBK-23M-F2 | gttctcgacgagtcAgctcatcggtca |  |
| pNBK-23M-R2 | AGTCCGTAATACGACTCACTTAAGACGTGACGGACCGGTTCCGT |  |
| pNBK-24M-F1 | GCGGCGGCCGCTCCGCGAGTAAGCTTatcgctgacgtattcgag | primers used for the *orf24*M strain, J7-1-F *orf24M* construction |
| pNBK-24M-R1 | cgtccgggcgagttgactgtacgatgtcg |  |
| pNBK-24M-F2 | cgacatcgtacagtcaactcgcccggacg |  |
| pNBK-24M-R2 | CAGTCCGTAATACGACTCACTTAAGgtctggttagagcgctcgg |  |
| pNBK-25M-F1 | GCGGCGGCCGCTCCGCGAGTAAGCTTctgtgtgcgtggctcgtc | primers used for the *orf25*M strain, J7-1-F *orf25M* construction |
| pNBK-25M-R1 | gacggtctcaccgtAgccgtcggtcgaca |  |
| pNBK-25M-F2 | tgtcgaccgacggcTacggtgagaccgtc |  |
| pNBK-25M-R2 | CCAGTCCGTAATACGACTCACTTAAGcgcgacggccgttgattc |  |
| pNBK-26M-F1 | GCGGCCGCTCCGCGAGTAAGCTTagcagcggagtgcgcca | primers used for the *orf26*M strain, J7-1-F *orf26M* construction |
| pNBK-26M-R1 | ataaccatgattcacgattaaacgaacgtagcagtacct |  |
| pNBK-26M-F2 | aggtactgctacgttcgtttaatcgtgaatcatggttat |  |
| pNBK-26M-R2 | CCAGTCCGTAATACGACTCACTTAAGgctgctttggcacacgggcg |  |
| 2260-F | acgaacccttgcaattgcttaagcaattgtcaatcacaga | primers used for the complementary of J7-1-FΔint::*pyrF* |
| 2260-R | aaacggggtggtcggcgtgagcatgcaagcttggcgtaatcatg |  |
| del12-13-F1 | tgtgggtctaccccacatttgcgatcatcgcgatcgttgc | Δ12-13 |
| del12-13-R1 | gcaacgatcgcgatgatcgcaaatgtggggtagacccaca |  |
| del12-17-F1 | tcgaacccgacgccctgtgcaacgcccgtg | Δ12-17 |
| del12-17-R1 | cacgggcgttgcacagggcgtcgggttcga |  |
| del11-17-R | tgctatgcatagctagcgcggccgcgggcgtcgggttcgaaa | Δ11-17 |
| del11-18-R | tgctatgcatagctagcgcggccgcatttttcgcgcgccccg | P3-19 |
| del11-18(-)-R | ctatgcatagctagcgcggccgcctatcggggcgcgat | P3-19(-) |
| del11-19-R | atgattacgccaagcttgcatgctcacagtaacccctccc | P3-20 |
| del11-20-R | gcatgctatgcatagctagcgcggccgcggttacgcacctccggcctc | P3-21 |
| del11-21-R | atgattacgccaagcttgcatgctcacgcccgatcaccta | P3-22 |
| pSNJ2-M-F | gtgacgacgatcaccctcTAAgggTaactcgcgactctccgtgcg | Integrase mutation |
| pSNJ2-M-R | cgcacggagagtcgcgagttAcccTTAgagggtgatcgtcgtcac |  |
| 26 crossover-F | aaccccttcggactgg | confirm the single-crossover integrants and double-crossover recombinants |
| 26 crossover-R | gagaacctgaacgaaatca |  |
| 25 crossover-F | tcaccgctggccttcgg |  |
| 25 crossover-R | cctcatctcggtctcgc |  |
| 23/24 crossover-F | atcacctaccgttaccgac |  |
| 23/24 crossover-R | acaattcgaggtgcgat |  |
| 22 crossover-F | tcagcaactcctccggc |  |
| 22 crossover-R | gtcgcgttttcggcgat |  |
| 21 crossover-F | tcagcaactcctccggc |  |
| 21 crossover-R | gcacacagaacacgggag |  |
| 20 crossover-F | gcgtcggtgacgttcga |  |
| 20 crossover-R | ggaatccgaggtcgcga |  |
| 19 crossover-F | gcgtcggtgacgttcga |  |
| 19 crossover-R | acctacttcggcgggc |  |
| ORF3-F | CCGACCCGATTCGACCCcaattgatgtacaacgataattc | ORF1-3 truncation |
| ORF2-F | CCGACCCGATTCGACCCcaattgatgaatgacgagttcct |  |
| ORF1-F | CCGACCCGATTCGACCCcaattggtggtgatcggaatgag |  |
| 19in-F | atctcctcggggtcga | *orf19* transcription (973 bp) |
| 19in-R | ggaaagaggctaagcactac |  |
| 20in-F | ctggttcaaccgcgtc | *orf20* transcription (150 bp) |
| 21in-R | gcgatcgcgtggaact | *orf21* transcription (303 bp) |
| 22in-R | gacgagcaacggcgat | *orf22* transcription (273 bp) |
| 25in-R | gccgtcggtcgacaat | *orf25* transcription (175 bp) |
| Q-19-F | GTAGTGCTTAGCCTCTTTCC | *orf19-20* co-transcription (1010 bp) |
| Q-20-R | gtggccgacctgacagcc |  |
| Q-20-F | acagtaacccctcccgtt | *orf20-21* co-transcription (679 bp) |
| Q-21-R | GGATGCAGTATTCGGAGTGG |  |
| Q-21-F | GCCTCCTGGACGGTCTTGT | *orf21-22* co-transcription (398 bp) |
| Q-22-R | GGGCGGGATGTACCTGTT |  |
| Q-22-F | CCTACCGTTACCGACGAATC | *orf22-23* co-transcription (624 bp) |
| Q-23-R | tgagccgactcgtcgaga |  |
| Q-23-F | gcgcgttccgaaccgcgc | *orf23-24* co-transcription (361 bp) |
| Q-24-R | CCATCAAGCAACCGACGAG |  |
| Q-24-F | GCCCAGTAGATGTAGATGAGTCC | *orf24-25* co-transcription (333 bp) |
| Q-25-R | cgcgttttcggcgatga |  |
| Q-25-F | ctctgggtccggaatcga | *orf25-26* co-transcription (408 bp) |
| Q-26-R | CGTAGCAGTACCTGTGGG |  |
| Q-26-F | gttcggttccagttcagtg | *orf26*-*int* co-transcription (561 bp) |
| Q-Int-R | cgacggaccgcgagata |  |
| Nest-F1 | gttcggttccagttcagtg | Primers used in nested-PCR |
| Nest-R1 | cggggccgattggga |  |
| Nest-F2 | gaaggtagtcgcggcg |  |
| Nest-R2 | gtcgcaagcgcgaacg |  |
| *mevR*-F | ttgtactgagagtgcaccatatgtcgagtcgtcccacgtg | *mevR*-pSNJ2Int^Mut^P3-19(+) Replace the antibiotic screening marker with mev |
| *mevR*-R | gcacagtctgtgattgacaattgagctcgaagagtggcat |  |
| Q-*attP*-F | CGACGCGAGCGATGATAT | primers used  in qPCR  assays  E=0.91 |
| Q-*attP*-R | GGGTTTGTTGCGGATGG |  |
| Q-*radA*-F | ATCCACGTCGCGAAGGCGTTCA | primers used  in qPCR  assays  E=0.92 |
| Q-*radA*-R | CTGCTTGTTGAGTTTCTGCTGTCGGTC |  |
| Q-ORF7-F | ATCTGTCGGAGTAACGGCTCG | primers used  in qPCR  assays  E=0.96 |
| Q-ORF7-R | GGGCGGCACTGGGTATTTG |  |
| Q-*attP*’-F | GGGTTTGTTGCGGATGG | primers used  in qPCR  assays  E=1 |
| Q-*attP*’-R | CGACTGAAGAAGCGACTCAA |  |
| *attP*-F | tcatcggtcacctcc | used for *attP*/*attP*′ detection |
| *attP*-R | ccgagaattggaccg |  |
| *attL-*F | ctgagtgatagtccgttccg | used for detecting the plasmid integration and circularization status |
| *attP-F2* | gcccagtagatgtagatgag |  |
| *attP-R2* | GCGGTGGAACAGTATCAGC |  |
| 1-656 bp-F | gggcgcgcgaaaaatgcggccgcacacgtagtatcgta | Validation of SNJ1 superinfection |
| 1-656 bp-R | gaccatgattacgccaagcttgagctcgcgatcgtt |  |
| ORF19-270 bp-R | tgctatgcatagctagcgcggccgcatttttcgcgcgccccg | The study of the conserved motif |
| ORF19-231 bp-R | tgctatgcatagctagcgcggccgcctatcgcggcggcaggg |  |
| ORF19-210 bp-R | tgctatgcatagctagcgcggccgctagtttgggatcttggc |  |
| ORF19-204 bp-R | tgctatgcatagctagcgcggccgcgggatcttggccttagc |  |
| ORF19-198 bp-R | tgctatgcatagctagcgcggccgcttggccttagccacacc |  |
| ORF19-192 bp-R | tgctatgcatagctagcgcggccgcttagccacaccccctaa |  |
| ORF19-180 bp-R | tgctatgcatagctagcgcggccgccctaacccggaattcga |  |
| ORF19-90 bp-R | tgctatgcatagctagcgcggccgcacgcgccgcgagcccgg |  |
| Hpro-*amyH*-F | cgcgaaaaatgcggccgcaattgtcttcgctgccgccgtt | The study of the expression activity of pSNJ2Int^Mut^P3-19(+) |
| Hpro-*amyH*-R | cttgcatgctatgcatagctagcCTAgtggtggtggtggtggtgCTCGGGTGCGTAGCAGA |  |
| *Mun*I-P-F | tcagacgaacccttgcaattgTAATCTCGTGGTATC | The study of the expression activity of pFJ6-P-ORF26-GFP |
| P-ORF26-R | atatcgtgaatcatTCAGGATCCCATCTCC |  |
| P-ORF26-F | GGAGATGGGATCCTGAatgattcacgatat |  |
| ORF26-GFP-F | aacggggtggtcggcgGGCGGCGGTGGGTCGGGCGGCGGTGGGTCGG |  |
| ORF26-GFP-R | CCGACCCACCGCCGCCCGACCCACCGCCGCCcgccgaccaccccgtt |  |
| *Sph*I-GFP-R | catgattacgccaagcttgcatgcTTATTTGTATAGTTCATC |  |
| P-19-F | GAGATGGGATCCTGAgttactgtgagcgcg | The study of the expression activity of pFJ6-P-ORF19-His |
| P-19-R | cgcgctcacagtaacTCAGGATCCCATCTC |  |
| *Afl*II-ORF19His-R | gcggccgcactagtcttaagctagtggtggtggtggtggtgtcggggcgcgatccca |  |
| SNJ2-TZ-F | gtgatcggggagctgaccga | Southern blot assay |
| SNJ2-TZ-R | gtcgacatacggaacgccga |  |
| E25A-F | cggtttcagtgccacgCgttcgacgggcacttt | Construction of conservative site mutation plasmids on pSNJ2Int^Mut^P3-19(+) |
| E25A-R | aaagtgcccgtcgaacGcgtggcactgaaaccg |  |
| D207A-F | acgtcgaacgtcaccgccgcggcagtgtacgtg |  |
| D207A-R | cacgtacactgccgcggcggtgacgttcgacgt |  |
| Y283A-F | aaagaggctaagcacGCctacatgaaggacccg |  |
| Y283A-R | cgggtccttcatgtagGCgtgcttagcctcttt |  |
| Y283F-F | aaagaggctaagcacTTctacatgaaggacccg |  |
| Y283F-R | cgggtccttcatgtagAAgtgcttagcctcttt |  |
| K281A-F | atcgggaaagaggctGCTcactactacatgaag |  |
| K281A-R | cttcatgtagtagtgAGCagcctctttcccgat |  |
| H282A-F | gggaaagaggctaagGCTtactacatgaaggac |  |
| H282A-R | gtccttcatgtagtaAGCcttagcctctttccc |  |
| Y284A-F | gaggctaagcactacGCTatgaaggacccggac |  |
| Y284A-R | gtccgggtccttcatAGCgtagtgcttagcctc |  |
| M285A-F | gctaagcactactacgctaaggacccggacaac |  |
| M285A-R | gttgtccgggtccttagcgtagtagtgcttagc |  |
| E303A-F | tggcatccaaaactcgCgatcggctaccagacg |  |
| E303A-R | cgtctggtagccgatcGcgagttttggatgcca |  |
| D408A-F | acgttgctcaccgacgCcggcgagatctcaccc |  |
| D408-R | tgagatctcgccgccgGcggtgagcaacgtgtc |  |
| G409A-F | acgttgctcaccgacgCcggcgagatctcaccc |  |
| G409A-R | gggtgagatctcgccgGcgtcggtgagcaacgt |  |
| G410A-F | ttgctcaccgacggcgCcgagatctcaccccag |  |
| G410A-R | ctggggtgagatctcgGcgccgtcggtgagcaa |  |
| 408-410A-F | gacacgttgctcaccgCcgCcgCcgagatctcaccccag |  |
| 408-410A-R | ctggggtgagatctcgGcgGcgGcggtgagcaacgtgtc |  |

**SUPPLEMENTARY FIGURES**

**Supplementary Figure 1. Genomic comparison of productive and integrase-encoding temperate viruses within the genus *Betapleolipovirus***

ORFs and genes are depicted as arrows, with the corresponding ORF/genes numbers indicated within the arrows. Homologous genes are color-coded for clarity. Pairwise amino acid identity (%) is shown between the sequences. Genes encoding structural proteins are labeled at the top. The image was generated using Easyfig version 2.2.3. Accession numbers for all genomes included in this comparison are indicated in the figure. SNJ2, N6C1A, N8T1, HHPV9, and HHPV4, which carry integrase genes, are temperate viruses belonging to the genus *Betapleolipovirus*. In contrast, HHPV3, HRPV3, HRPV10, HRPV11, HRPV12, HRPV13, HGPV1 are productive betapleolipoviruses isolated from the environment.

**Supplementary Figure 2. Excision and circularization of the SNJ2 provirus enables its terminal regions to form a novel, functional integrase-containing operon that defines the viral replication region**

a. The upper panel illustrates the gene arrangement of SNJ2 in its integrated state, with the host genome depicted in blue and dashed lines indicating omitted segments of the genome. The lower map shows the arrangement of the viral genome following excision and circularization. Arrows represent open reading frames (ORFs) and their transcriptional orientation, with the orange arrow highlighting the newly formed ORF26 after circularization. The transcription start position may be located within *orf1* (419 bp upstream of the stop codon) and the end site may be located 50 bp downstream *orf19*.

b. Western blot analysis detected the ORF26-GFP fusion protein, with a band size of approximately 35.1 kDa. The J7-1-F/pFJ6-ORF26-GFP were cultured to middle exponential phase (OD_600_=0.6-0.8) in H-2. Samples were collected, and crude extracts were subjected to SDS-PAGE. Lanes 1 and 2 represent biological replicates of the sample. Western blot analysis was performed using an anti-GFP antibody.

c. *Orf1* and *orf26*-*orf19* form an operon with the transcription start site located inside *orf*1. For co-transcription analysis, the primer pairs used and the expected sizes of the amplified junction fragments are indicated as follows: *orf19* to *orf20* (Q-19-F/Q-20-R, 1010 bp), *orf20* to *orf21* (Q-20-F/Q-21-R, 679 bp), *orf21* to *orf22* (Q-21-F/Q-22-R, 398 bp), *orf22* to *orf23* (Q-22-F/Q-23-R, 624 bp), *orf23* to *orf24* (Q-23-F/Q-24-R, 361 bp), *orf24* to *orf25* (Q-24-F/Q-25-R, 333 bp), *orf25* to *orf26* (Q-25-F/Q-26-R, 408 bp), *orf26* to *orf1* (Q-26-F/Q-Int-R, 561 bp), *orf19* to *orf25* (Q-19-F/Q-25-R, 2742 bp), *orf19* to *Int* (Q-19-F/Q-Int-R, 3358 bp). Lanes: +, positive control with J7-1 genomic DNA as the template; −, negative control using total RNA as the template; RT, RT (Reverse Transcription)-PCR with cDNA as the template; M, DNA marker (Trans 2K plus II).

d. Standard PCR analysis of *attP* site formation, a marker for viral genome circularization, at 3 hours post-MMC induction. The analysis was performed on the parental strain (J7-1-F), a catalytically inactive integrase mutant (Y330A), and mutants within the replication operon (*orf19*M to *orf26*M).

e. Strains (as above) and a capsid gene deletion mutant (Δ*orf*13) were cultured to mid-exponential phase (OD_600_=0.6-0.8) in Halo-2 medium and treated with MMC (1μg /mL) for 30 min as described previously. Samples were collected every three hours post-induction and the *attP* site of the SNJ2 genome was quantified by qPCR using the primer pairs *attP*-F/ *attP*-R. The bars represent the means and standard deviations of three independent experiments.

**Supplementary Figure 3.** **The flowchart of pSNJ2Int^Mut^, pSNJ2Int^Mut^ P3-19(+) and *mevR*-pSNJ2Int^Mut^ P3-19(+) construction.**

a/b. Schematic diagram of pSNJ2Int^Mut^, pSNJ2Int^Mut^ P3-19(+) and *mevR*-pSNJ2IntMut P3-19(+): The plasmid contains the following key elements: Ampicillin resistance (*AmpR*, Red), Uracil biosynthesis gene (*pyrF*, pink) or Mev resistance gene (*mevR*, blue), ColE1 origin (*ori*), SNJ2 replication region (P3-ORF11) and the multiple cloning site (MCS) flanked by unique restriction sites (*Not*I*, Nhe*I*, Bmt*I*, Hind*III). Arrows indicate transcriptional orientation.

**Supplementary Figure 4. Expression activity and host range of the shuttle vector**

a. PCR analysis of the *attP* (attP-F2/attP-R2,1085 bp) and *attL* (attL-F/attP-R2, 707 bp) integration junctions. Colony PCR was performed on archaeal transformants to assess potential site-specific chromosomal integration. Lanes 1-3: Three independent transformants harboring the pSNJ2Int^Mut^ plasmid. Lanes 4-6: Three independent transformants harboring the pSNJ2Int^Mut^P3-19(+) plasmid. The absence of specific integration bands confirms that the plasmids do not integrate via the integrase *att* system and are maintained episomally.

b. Plasmid rescue and restriction digestion profiling. Total DNA was extracted from the archaeal transformants, back-transformed into *E. coli*, and the rescued plasmids were subjected to restriction enzyme digestion (*Stu*I and *Hind*III) to verify their structural integrity. Odd-numbered lanes (1, 3, and 5) represent the restriction profiles of the originally constructed shuttle vectors, while even-numbered lanes (2, 4, and 6) represent the profiles of the corresponding rescued plasmids. Lanes 1 and 2: pSNJ2^Int^Mut (9357 bp, 6796 bp); Lanes 3 and 4: pSNJ2Int^Mut^P3-19(+) (9357 bp, 685 bp); Lanes 5 and 6: pSNJ2P-19(+) (5514 bp, 685 bp). The identical restriction banding patterns between the original and rescued plasmids in each paired comparison strictly demonstrate that these shuttle vectors replicate autonomously as intact, free-form circular DNA molecules without undergoing chromosomal recombination or structural rearrangements. The plasmid rescue and restriction digestion assays were performed in three independent biological replicates, yielding consistent results. A representative gel image is shown.

c/d. Transformants containing both pYC-SHS/ pSNJ2Int^Mut^ P3-19(+) (c) or pFJ6-MCS/*mevR*-pSNJ2Int^Mut^ P3-19(+) (d) were selected on 20% MM plates supplemented with 5 μg/mL mevinolin. Positive transformants were cultured in 20% MM liquid medium with Mev, followed by antibiotic-free Halo-2 medium. Quantitative PCR (qPCR) using *pyrF*/*radA* and *mevR*/*radA* primer sets was performed to measure relative plasmid copy numbers after antibiotic-free subculturing. The bars represent the means and standard deviations of three independent experiments. Note: G0-G4 denote Generation 0 to Generation 4, representing successive rounds of subculturing during the stability assay.

e. Amylase expression in J7-3-F cells. pSNJ2Int^Mut^ P3-19(+)-*amyH* was transformed into J7-3-F cells. Twelve random transformants were transferred to 5 ml of 20% MM without uracil and grown to log phase. Aliquots (2 μL) were spotted onto 20% MM plates supplemented with 2% (wt/vol) soluble starch. After 2 days, iodine solution was added to the plates, and haloes formed around the selected transformants, indicating amylase expression. N1, J7-3-F cultures (negative control); N2, J7-3-F transformed with pSNJ2Int^Mut^ P3-19(+) (negative control); 1 to 12, transformants of J7-3-F harboring pSNJ2Int^Mut^ P3-19(+)-*amyH*.

f. Expression of SNJ1-*orf4*(1-656) dramatically inhibited plaque formation. Tenfold serial dilutions of SNJ1 viruses were spotted onto the lawns of CJ7-F/ pSNJ2Int^Mut^ P3-19(+), or CJ7-F/pFJ6-1-656, CJ7-F/1-656-pSNJ2Int^Mut^ P3-19(+). Plates were incubated at 37°C for 48 h and photographed. The maximum dilution for observed plaques is highlighted by asterisk.

 **Supplementary Figure 5.** **Predicted secondary structures of the conserved downstream region in *Betapleolipoviruses*.**

**Supplementary Figure 6. Mutational analysis of the ORF19 downstream sequence.**

a. Mutational analysis of the conserved stem-loop (176-197 nt). Schematic of the predicted conserved stem-loop structure, illustrating the locations and predicted structural consequences of the introduced point mutations: M1 (G188C, C195G, structure-preserving), M2 (A191T, A192T, loop-mutation), M3 (T186G, G187T, stem-disrupt), and M4 (C195A, C196A, stem-disrupt). The predicted structure is color-coded based on sequence conservation: completely conserved bases are shown in purple and highly conserved bases are in blue. The predicted stem-loop structure formed by the upstream IR (152-166 nt) is highlighted in red. Other less conserved regions that do not form secondary structures are shown in black. Mutated bases are labeled in bold green font and shaded in yellow. Other less conserved regions that do not form secondary structures are shown in black.

b. Mutational analysis of the upstream IR (152-166 nt). Schematic of the predicted stem-loop structure formed by the upstream IR (152-166 nt). The locations of mutations IRM1 (C156G, C157G, G161C, G162C, symmetry-preserving) and IRM2 (C156T, C157T, symmetry-disrupting) and IRM3 (G161A, G162A, G163A, T164C, symmetry-disrupting) are indicated. The color-coding scheme is identical to that described above.

**Supplementary Figure 7. Multiple sequence alignment of ORF19 (RepSNJ2) homologous proteins.**

The alignment includes ORF19 (RepSNJ2) homologs from 12 viruses of the *Betapleolipovirus* genus. Key functional sites are highlighted: the catalytic tyrosine (first red dashed box), conserved acidic residues (red asterisks), and conserved residues within the C-terminal DNA-binding domain (second red dashed box).

**Supplementary Figure S8. Western blot analysis of the wild-type and mutant ORF19 proteins.** The wild-type ORF19-His fusion protein (expected molecular weight: ~75 kDa) and its specific variants (E25A, D207A, Y283A, Y283F, E303A, G410A, and D408A/G409A/G410A) were expressed in the *Natrinema* sp. J7-3-F strain using the pFJ6-based overexpression system. The negative control lane contains protein extract from the J7-3-F/pFJ6-P-His strain, which expresses the His-tag alone without the ORF19 coding sequence. All samples were detected using an anti-His antibody. The lower panel shows the corresponding Ponceau S-stained membrane, serving as the total protein loading control.

**Supplementary Figure 9. Superposition of AlphaFold3-predicted structures of distantly related Rep-Arvir homologs (identified via iterative homology searches) with the ORF19 (RepSNJ2) structure (gold).** The conserved tyrosine catalytic site is highlighted in red.

 **Supplementary Figure 10. Structural conservation of the downstream motif associated with *rep-arvir* loci in viruses and plasmids within *Halobacteria*.**

**References**:

1. Bitan-Banin,G., Ortenberg,R. and Mevarech,M. (2003) Development of a gene knockout system for the halophilic archaeon Haloferax volcanii by use of the pyrE gene. *J Bacteriol*, **185**, 772–778.

2. Wang,J., Liu,Y., Liu,Y., Du,K., Xu,S., Wang,Y., Krupovic,M. and Chen,X. (2018) A novel family of tyrosine integrases encoded by the temperate pleolipovirus SNJ2. *Nucleic Acids Res*, **46**, 2521–2536.

3. Cai,S., Cai,L., Zhao,D., Liu,G., Han,J., Zhou,J. and Xiang,H. (2015) A novel DNA-binding protein, PhaR, plays a central role in the regulation of polyhydroxyalkanoate accumulation and granule formation in the haloarchaeon haloferax mediterranei. *Appl. Environ. Microbiol.*, **81**, 373–385.

4. Wang,Y., Sima,L., Lv,J., Huang,S., Liu,Y., Wang,J., Krupovic,M. and Chen,X. (2016) Identification, Characterization, and Application of the Replicon Region of the Halophilic Temperate Sphaerolipovirus SNJ1. *J Bacteriol*, **198**, 1952–1964.

5. Lv,J., Wang,S., Zeng,C., Huang,Y. and Chen,X. (2013) Construction of a shuttle expression vector with a promoter functioning in both halophilic Archaea and Bacteria. *FEMS Microbiol Lett*, **349**, 9–15.

6. Zhang,Z., Liu,Y., Wang,S., Yang,D., Cheng,Y., Hu,J., Chen,J., Mei,Y., Shen,P., Bamford,D.H., *et al.* (2012) Temperate membrane-containing halophilic archaeal virus SNJ1 has a circular dsDNA genome identical to that of plasmid pHH205. *Virology*, **434**, 233–241.

7. Liu,Y., Wang,J., Liu,Y., Wang,Y., Zhang,Z., Oksanen,H.M., Bamford,D.H. and Chen,X. (2015) Identification and characterization of SNJ2, the first temperate pleolipovirus integrating into the genome of the SNJ1-lysogenic archaeal strain. *Mol Microbiol*, **98**, 1002–1020.

8. Wang,Y., Chen,B., Sima,L., Cao,M. and Chen,X. (2017) Construction of Expression Shuttle Vectors for the Haloarchaeon Natrinema sp. J7 Based on Its Chromosomal Origins of Replication. *Archaea*, **2017**, 4237079.

9. Chen,Z., Liu,Y., Wang,Y., Du,X., Deng,X., Xiang,J., Wang,Y., Wang,J., Krupovic,M., Du,S., *et al.* (2023) A virus-borne DNA damage signaling pathway controls the lysogeny-induction switch in a group of temperate pleolipoviruses. *Nucleic Acids Res*, **51**, 3270–3287.

10. Chen,B., Chen,Z., Wang,Y., Gong,H., Sima,L., Wang,J., Ouyang,S., Gan,W., Krupovic,M., Chen,X., *et al.* (2020) ORF4 of the Temperate Archaeal Virus SNJ1 Governs the Lysis-Lysogeny Switch and Superinfection Immunity. *J Virol*, **94**, e00841-20.

11. Xiang,J., Sonani,R.R., Wang,Y., Chen,Z., Xiong,W., Chen,J., Li,S., An,K., Wang,Y., Liu,Y., *et al.* (2025) A type IV pili-mediated mutualism between two co-resident temperate archaeal viruses and their host. *Cell Reports*, **44**.

12. Allers,T., Ngo,H.-P., Mevarech,M. and Lloyd,R.G. (2004) Development of additional selectable markers for the halophilic archaeon Haloferax volcanii based on the leuB and trpA genes. *Appl Environ Microbiol*, **70**, 943–953.
